# An efficient direct screening system for microorganisms that activate plant immune responses based on plant–microbe interactions using cultured plant cells

**DOI:** 10.1038/s41598-021-86560-0

**Published:** 2021-04-01

**Authors:** Mari Kurokawa, Masataka Nakano, Nobutaka Kitahata, Kazuyuki Kuchitsu, Toshiki Furuya

**Affiliations:** Department of Applied Biological Science, Faculty of Science and Technology, Tokyo University of Science, 2641, Yamazaki, Noda, Chiba 278-8510 Japan

**Keywords:** Applied microbiology, Bacteria, Plant immunity

## Abstract

Microorganisms that activate plant immune responses have attracted considerable attention as potential biocontrol agents in agriculture because they could reduce agrochemical use. However, conventional methods to screen for such microorganisms using whole plants and pathogens are generally laborious and time consuming. Here, we describe a general strategy using cultured plant cells to identify microorganisms that activate plant defense responses based on plant–microbe interactions. Microbial cells were incubated with tobacco BY-2 cells, followed by treatment with cryptogein, a proteinaceous elicitor of tobacco immune responses secreted by an oomycete. Cryptogein-induced production of reactive oxygen species (ROS) in BY-2 cells served as a marker to evaluate the potential of microorganisms to activate plant defense responses. Twenty-nine bacterial strains isolated from the interior of *Brassica rapa* var. *perviridis* plants were screened, and 8 strains that enhanced cryptogein-induced ROS production in BY-2 cells were selected. Following application of these strains to the root tip of *Arabidopsis* seedlings, two strains, *Delftia* sp. BR1R-2 and *Arthrobacter* sp. BR2S-6, were found to induce whole-plant resistance to bacterial pathogens (*Pseudomonas syringae* pv. *tomato* DC3000 and *Pectobacterium carotovora* subsp. *carotovora* NBRC 14082). Pathogen-induced expression of plant defense-related genes (*PR-1*, *PR-5*, and *PDF1.2*) was enhanced by the pretreatment with strain BR1R-2. This cell–cell interaction-based platform is readily applicable to large-scale screening for microorganisms that enhance plant defense responses under various environmental conditions.

## Introduction

Plants have evolved unique immune responses to protect against a variety of pathogens^1,2^. Plants perceive pathogen invasion via interactions between pattern recognition receptors on the cell surface and conserved molecular signature molecules known as pathogen/microbe-associated molecular patterns (PAMPs/MAMPs). Following pathogen recognition, a series of defense responses is induced, collectively known as PAMP-triggered immunity (PTI). Over time, however, specific pathogens have acquired the ability to suppress PTI in plants. These pathogens secrete various PTI-interfering effectors in the host plants. However, if the host plants acquire the ability to recognize these effectors via R (resistance) proteins, effector-triggered immunity (ETI) is induced, which involves stronger and longer-lasting responses than PTI. Early defense responses common to PTI and ETI include an increase in cytosolic Ca^2+^ concentration, production of reactive oxygen species (ROS), activation of the mitogen-activated protein kinases (MAPKs), expression of various defense-related genes, and increased biosynthesis of phytoalexins and defense hormones, such as salicylic acid (SA) and jasmonic acid (JA)^3–5^.

In addition to these local defense responses, plants exhibit systemically induced defense responses collectively known as systemic acquired resistance (SAR)^6^. In SAR, when pathogen-stimulated plants are subsequently challenged by pathogens, they exhibit more rapid and/or stronger activation of defense responses that enable them to resist to a wide range of pathogens. For example, inoculation of *Arabidopsis thaliana* with the pathogenic bacterium *Pseudomonas syringae* pv. *tomato* triggers SAR, which in turn induces resistance to the pathogenic oomycete *Peronospora parasitica*^7^.

In addition to pathogen-induced SAR, non-pathogenic microorganisms can induce systemic defense responses known as induced systemic resistance (ISR)^8^. For example, the rhizobacterium *Pseudomonas fluorescens* WCS417r can trigger ISR in several plant species, including *Arabidopsis* and carnation^9,10^. Pretreatment of *Arabidopsis* with *Streptomyces* sp. strain EN27, an endophytic actinobacterium isolated from wheat, enhances resistance to the bacterial pathogen *Pectobacterium carotovora* subsp. *carotovora* and fungal pathogen *Fusarium oxysporum*^11^. The well-known beneficial bacterium *Paraburkholderia phytofirmans* PsJN can induce *Arabidopsis* resistance to *P. syringae* pv. *tomato* through ISR^12,13^.

Microorganisms that activate plant immune responses have a high potential for application as biocontrol agents in agriculture because they could reduce the demand for pesticides^14,15^. Such microorganisms have attracted considerable attention because they function like vaccines without producing undesirable effects (e.g., growth inhibition) in plants^8,15^. Endophytes are particularly well suited for use as biocontrol agents due to their inherent ability to stably colonize the interior of plants. However, conventional methods to screen for such microorganisms use whole plants and pathogens and thus tend to be laborious and time consuming^9–11^, resulting in the identification of few microorganisms that activate plant defense responses. Moreover, microorganisms that produce antimicrobial compounds or exclude pathogens via niche competition can also be selected using conventional methods based on observation of disease symptoms. Therefore, these conventional methods are not suitable for direct screening for microorganisms that activate plant defense responses.

Cultured plant cells are useful as a simplified experimental system for studying plant immunity^16^. Tobacco BY-2 cells, which exhibit rapid and stable growth, are typical cultured plant cells^17^. Cryptogein, a proteinaceous elicitor of plant immune responses produced by the pathogenic oomycete *Phytophthora cryptogea*, is a well-studied model useful for elucidating the mechanisms of plant defense responses in BY-2 cells ^18–23^. Upon recognizing cryptogein, BY-2 cells exhibit plasma membrane ion fluxes, an increase in cytosolic Ca^2+^ concentration, and NADPH oxidase–dependent ROS production^19^. These initial responses are accompanied by activation of MAPKs and accumulation of defense-related gene transcripts^21,22^. Cryptogein-induced ROS production is closely correlated with the expression of defense-related genes and hypersensitive cell death; thus, it could be a suitable marker for evaluating defense responses in BY-2 cells^18,21,22^. These findings suggest that monitoring cryptogein-induced ROS production in BY-2 cells is a suitable experimental system for screening for microorganisms or chemicals that activate plant immune responses.

In this study, we describe a system based on plant–microbe interactions through physical and chemical signals for exploring the potential of microorganisms to activate plant immune responses. We monitored cryptogein-induced ROS production in BY-2 cells as an efficient marker to identify microorganisms capable of activating plant defense responses. This system involves incubation of a microorganism with tobacco BY-2 cells, followed by treatment with cryptogein and quantitative detection of ROS production via chemiluminescence. Our system streamlines the process of screening for microorganisms that “prime” and potentiate plant immune responses, thus helping plants resist pathogens.

We first isolated bacteria from *Brassica rapa* var. *perviridis* as model microorganisms. A total of 31 bacterial strains isolated from the plant interior were assayed using the screening system, and strains that enhanced cryptogein-induced ROS production in BY-2 cells were selected. We identified two novel endophytes that induce bacterial pathogen resistance in whole *Arabidopsis* plants. This cell–cell interaction–based platform could facilitate the discovery of plant immunity–activating microorganisms from a variety of sources.

## Results

### Isolation of bacteria from the interior of *B. rapa* var. *perviridis*

**Figure 1:**
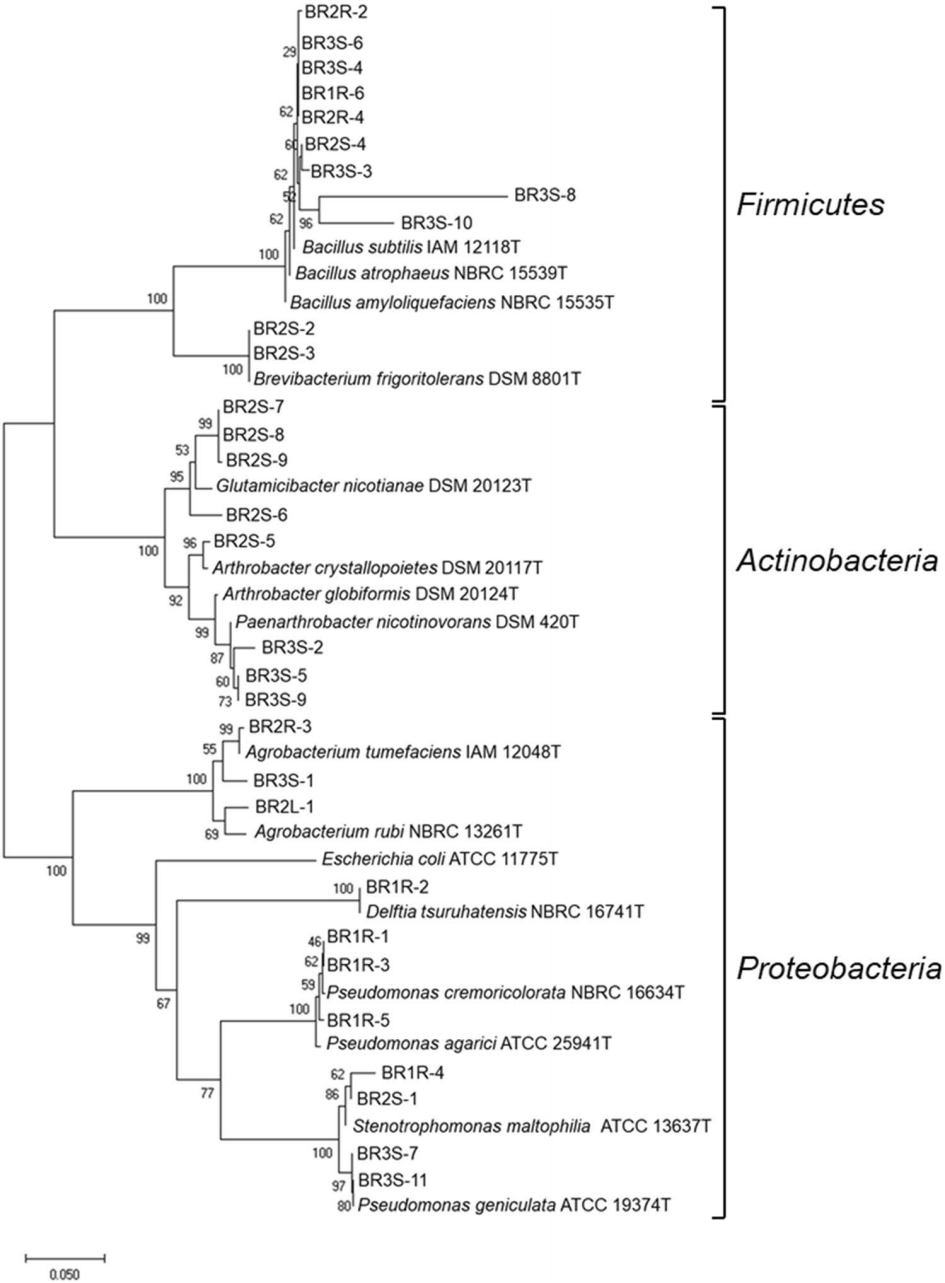
Phylogenetic relationships of bacterial strains recovered from the interior of *B. rapa* var. *perviridis* based on the 16S rDNA sequence. The bootstrap values from 1000 replications are shown at each of the branch points on the tree. Strain BR2R-1 is not included in the phylogenetic tree, because the 16S rDNA sequence contains an insertion (ca. 300 bp).

We isolated bacteria from the interior of *B. rapa* var. *perviridis* grown by organic farming without the use of pesticides. Roots, stems, and leaves were cut into small pieces and surface-sterilized using appropriate concentrations of sodium hypochlorite and ethanol^24^, as described in the Materials and Methods. After surface-sterilization, each tissue sample was rinsed with sterile water and placed on NBRC802 and ISP2 agar plates. The water used for rinsing was also spread onto each medium as a control. When no microorganisms appeared on medium for the control, that is, the surface had been sterilized, colonies that formed around the tissues were selected as putative endophytes. Using this isolation procedure, a total of 31 bacterial strains were isolated, of which 10 and 20 strains were derived from roots and stems, respectively, and 1 strain was derived from a leaf (Table 1). Taxonomic identification of these strains was performed based on 16S rDNA sequencing, and a phylogenetic tree of the sequences was constructed (Fig. 1). We found a variety of cultivable bacteria in the microbiome. These bacteria belonged to 9 different genera: *Bacillus*, *Brevibacterium*, *Glutamicibacter*, *Arthrobacter*, *Paenarthrobacter*, *Agrobacterium*, *Delftia*, *Pseudomonas*, and *Stenotrophomonas* (Table 1 and Fig. 1). Four strains (BR2L-1, BR3S-2, BR3S-8, and BR3S-10) exhibited low identity (< 96%) to previously reported sequences of typical strains, indicating that these strains might constitute new genera or species. The isolated bacteria were divided into 3 phyla, *Firmicutes*, *Actinobacteria*, and *Proteobacteria* (Fig. 1). Excluding potential human pathogenic bacteria (two *Stenotrophomonas* strains), the isolated bacteria were analyzed further.

**Table 1:** Bacterial strains recovered from the interior of *B. rapa* var. *perviridis*.

Strain	Host tissue	Phylum	Accession no	Closest type strain (accession no.)	Similarity (%)
BR1R-1	Root	*Proteobacteria*	LC511706	*Pseudomonas cremoricolorata* NBRC 16634 T (NR_113855.1)	571 / 576 (99)
BR1R-2	Root	*Proteobacteria*	LC511707	*Delftia tsuruhatensis* NBRC 16741 T (AB681119)	878 / 878 (100)
BR1R-3	Root	*Proteobacteria*	LC511708	*Pseudomonas cremoricolorata* NBRC 16634 T (NR_113855.1)	501 / 504 (99)
BR1R-4	Root	*Proteobacteria*	LC511709	*Stenotrophomonas maltophilia* ATCC 13637 T (NR_112030.1)	449 / 469 (96)
BR1R-5	Root	*Proteobacteria*	LC511710	*Pseudomonas agarici* ATCC 25941 T (NR_115608.1)	417 / 425 (98)
BR1R-6	Root	*Firmicutes*	LC511711	*Bacillus atrophaeus* NBRC 15539 T (NR_112723.1)	446 / 451 (99)
BR2R-1	Root	*Proteobacteria*	LC511712	*Agrobacterium tumefaciens* IAM 12048 T (NR_041396.1)	852 / 864 (99)
BR2R-2	Root	*Firmicutes*	LC511713	*Bacillus atrophaeus* NBRC 15539 T (NR_112723.1)	600 / 608 (99)
BR2R-3	Root	*Proteobacteria*	LC511714	*Agrobacterium tumefaciens* IAM 12048 T (NR_041396.1)	550 / 553 (99)
BR2R-4	Root	*Firmicutes*	LC511715	*Bacillus atrophaeus* NBRC 15539 T (NR_112723.1)	678 / 681 (99)
BR2S-1	Stem	*Proteobacteria*	LC511716	*Stenotrophomonas maltophilia* ATCC 13637 T (NR_112030)	426 / 434 (98)
BR2S-2	Stem	*Actinobacteria*	LC511717	*Brevibacterium frigoritolerans* DSM 8801 T (NR_115064)	591 / 593 (99)
BR2S-3	Stem	*Actinobacteria*	LC511718	*Brevibacterium frigoritolerans* DSM 8801 T (NR_115064)	725 / 726 (99)
BR2S-4	Stem	*Firmicutes*	LC511719	*Bacillus atrophaeus* NBRC 15539 T (NR_112723.1)	554 / 561 (99)
BR2S-5	Stem	*Actinobacteria*	LC511720	*Arthrobacter crystallopoietes* DSM 20117 T (NR_026189.1)	609 / 613 (99)
BR2S-6	Stem	*Actinobacteria*	LC511721	*Arthrobacter globiformis* DSM 20124 T (NR_026187.1)	676 / 700 (97)
BR2S-7	Stem	*Actinobacteria*	LC511722	*Glutamicibacter nicotianae* DSM 20123 T (NR_026190.1)	839 / 852 (98)
BR2S-8	Stem	*Actinobacteria*	LC511723	*Glutamicibacter nicotianae* DSM 20123 T (NR_026190.1)	592 / 602 (98)
BR2S-9	Stem	*Actinobacteria*	LC511724	*Glutamicibacter nicotianae* DSM 20123 T (NR_026190.1)	606 / 618 (98)
BR2L-1	Leaf	*Proteobacteria*	LC511725	*Agrobacterium rubi* NBRC 13261 T (NR_113608.1)	443 / 467 (95)
BR3S-1	Stem	*Proteobacteria*	LC511726	*Agrobacterium tumefaciens* IAM 12048 T (NR_041396.1)	539 / 553 (97)
BR3S-2	Stem	*Actinobacteria*	LC511727	*Arthrobacter globiformis* DSM 20124 T (NR_026187.1)	462 / 494 (94)
BR3S-3	Stem	*Firmicutes*	LC511728	*Bacillus atrophaeus* NBRC 15539 T (NR_112723.1)	453 / 462 (98)
BR3S-4	Stem	*Firmicutes*	LC511729	*Bacillus atrophaeus* NBRC 15539 T (NR_112723.1)	556 / 561 (99)
BR3S-5	Stem	*Actinobacteria*	LC511730	*Paenarthrobacter nicotinovorans* DSM 420 T (NR_026194.1)	543 / 546 (99)
BR3S-6	Stem	*Firmicutes*	LC511731	*Bacillus atrophaeus* NBRC 15539 T (NR_112723.1)	530 / 532 (99)
BR3S-7	Stem	*Proteobacteria*	LC511732	*Pseudomonas geniculata* ATCC19374T (NR_024708.1)	482 / 487 (99)
BR3S-8	Stem	*Firmicutes*	LC511733	*Bacillus subtilis* IAM 12118 T (NR_112116.2)	381 / 435 (88)
BR3S-9	Stem	*Actinobacteria*	LC511734	*Paenarthrobacter nicotinovorans* DSM 420 T (NR_026194.1)	676 / 679 (99)
BR3S-10	Stem	*Firmicutes*	LC511735	*Bacillus amyloliquefaciens* NBRC 15535 T (NR_041455.1)	548 / 585 (94)
BR3S-11	Stem	*Proteobacteria*	LC511736	*Pseudomonas geniculata* ATCC 19374 T (NR_024708.1)	477 / 481 (99)

### ROS production induced by interaction between bacteria and cultured plant cells

We first examined interactions between the isolated bacteria and cultured plant cells by monitoring ROS production. Although microbial components such as lipopolysaccharides and several metabolites reportedly induce ROS production in cultured plant cells^25,26^, few reports have examined ROS production induced by intact microbial cells^27^. Tobacco BY-2 cells were incubated with each strain of isolated bacteria, and ROS production was monitored using a chemiluminescence assay with luminol. Most of the bacteria (19 strains) had no effect on BY-2 cells during co-incubation, based on ROS production (Fig. S1a). Interestingly, however, 10 strains (BR1R-2, BR1R-5, BR2R-4, BR2S-3, BR2S-6, BR3S-3, BR3S-7, BR3S-8, BR3S-10, and BR3S-11) induced ROS production after approximately 80 min of co-incubation (Fig. S1b). These results suggest that intact bacteria can induce ROS production by plant cells via interactions.

**Figure 2:**
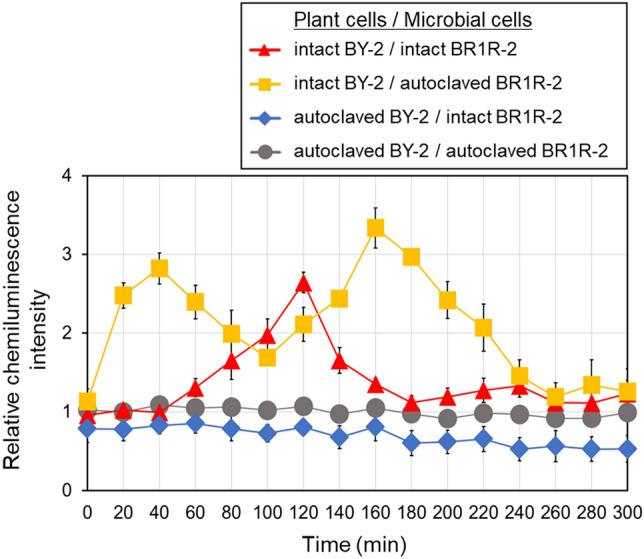
Time course of ROS production in BY-2 cells co-incubated with BR1R-2 cells. Intact BY-2 cells were co-incubated with intact BR1R-2 cells (∆) or autoclaved BR1R-2 cells (□). In another experiment, autoclaved BY-2 cells were co-incubated with intact BR1R-2 cells (◊) or autoclaved BR1R-2 cells (○). ROS production was monitored by chemiluminescence. The average value of the autoclaved BY-2/autoclaved BR1R-2 (○) sample was expressed as 1.0. Average values ± SE from three independent experiments are presented.

In order to determine whether the ROS was produced by the bacteria or cultured plant cells, the assays were repeated using cells killed by autoclave treatment. Incubation of autoclaved plant cells with intact *Delftia* sp. BR1R-2 cells resulted in no detectable ROS production. In contrast, incubation of intact plant cells with autoclaved bacteria resulted in a biphasic increase in ROS production. The first peak in ROS generation occurred after 40 min and was followed by a second peak that reached a maximum at approximately 160 min (Fig. 2), resembling the temporal pattern of cryptogein-triggered ROS production in tobacco BY-2 cells^18,19^. These results clearly demonstrate that bacteria act on BY-2 cells to induce ROS production. It is interesting to note that co-incubation with intact bacteria resulted in only one peak in ROS production, whereas co-incubation with autoclaved bacteria resulted in a biphasic increase in ROS. During co-incubation with intact strain BR1R-2 cells, some factor(s) derived from the bacteria might have scavenged ROS produced by the BY-2 cells. Considered collectively, these data indicate that this experimental system is useful for evaluating interactions between bacteria and cultured plant cells.

### Screening for microorganisms that prime plant immune responses based on plant–microbe interactions using cultured plant cells

We established an experimental system using intact bacteria and cultured plant cells to screen for microorganisms that prime plant immune responses. Cryptogein-induced ROS production in tobacco BY-2 cells was employed as a marker for the screening (Fig. S2). Buffer containing BY-2 cells was inoculated with culture solution of each isolated bacterial strain and incubated for 4 h. After the co-incubation, the cells were collected and suspended in fresh buffer to remove ROS scavengers and other bacteria-derived metabolites. Cryptogein, as an elicitor of plant immune responses, was then added to the buffer, and ROS production was monitored by chemiluminescence.

**Figure 3:**
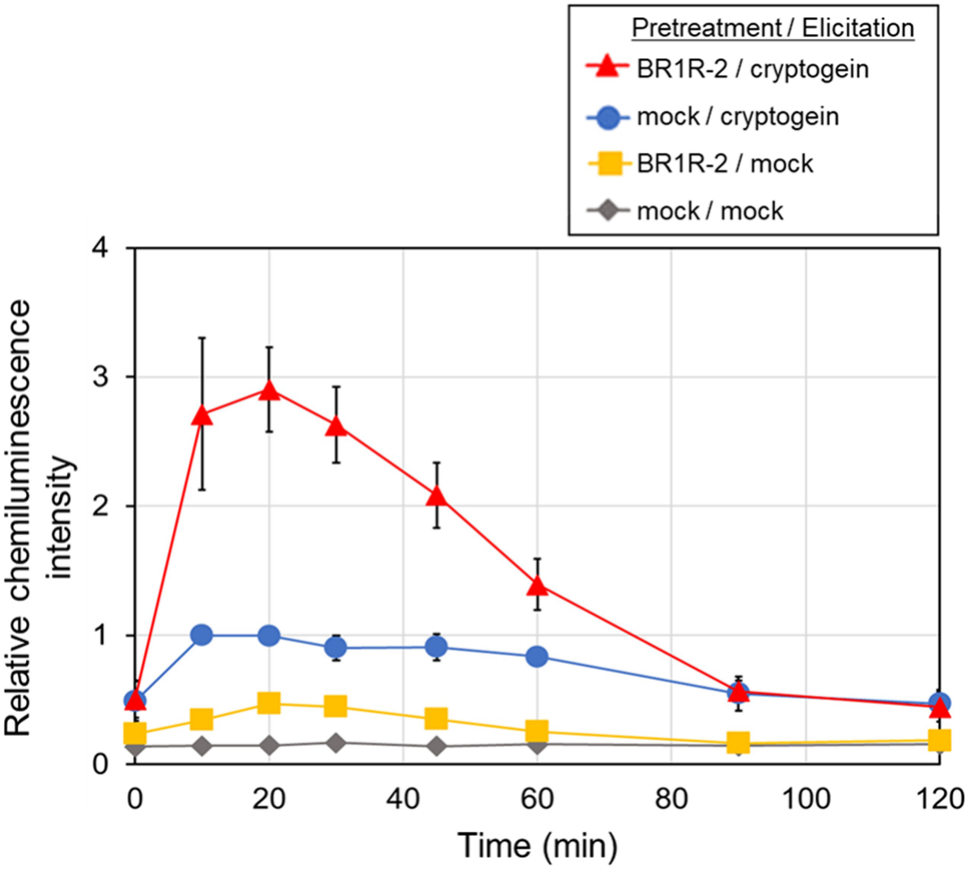
Time course of cryptogein-induced ROS production in BY-2 cells co-incubated with BR1R-2 cells. BY-2 cells were co-incubated with BR1R-2 cells (∆) or mock treatment (only a mixture of the medium and the buffer, ○), and then cryptogein was added. In another experiment, BY-2 cells were co-incubated with BR1R-2 cells (□) or mock treatment (only the mixture, ◊), and then mock elicitor (only the buffer) was added instead of cryptogein. ROS was monitored by chemiluminescence. The maximum value of the mock/cryptogein (○) sample was expressed as 1.0. Average values ± SE from three independent experiments are presented.

We used *Delftia* sp. BR1R-2 to validate the screening system (Fig. 3). Incubation of only plant cells or bacteria resulted in low ROS production after cryptogein addition. In contrast, pre-incubation of BY-2 cells with BR1R-2 cells resulted in greatly enhanced cryptogein-induced ROS production. The amount of ROS produced by BY-2 cells after BR1R-2 treatment was three times that produced by BY-2 cells after mock treatment. These results indicate that strain BR1R-2 is suitable for priming the immune responses of BY-2 cells.

This system was then used to screen for the plant immunity–activating potential of the other bacteria. Although most of the bacteria (21 strains) had no effect on BY-2 cells with co-incubation (Fig. S3a), 7 strains in addition to *Delftia* sp. BR1R-2 enhanced the cryptogein-induced ROS production (Fig. S3b): *Pseudomonas* sp. BR1R-3, *Pseudomonas* sp. BR1R-5, *Bacillus* sp. BR2R-4, *Bacillus* sp. BR2S-4, *Arthrobacter* sp. BR2S-6, *Agrobacterium* sp. BR3S-1, and *Paenarthrobacter* sp. BR3S-9. Interestingly, these immunity-inducing bacteria formed distinct phylogenetic clusters (Fig. 1). In addition, *P. phytofirmans* PsJN, a well-known biocontrol bacterium^12,13^, enhanced the cryptogein-induced ROS production (Fig. S4), although the amount of ROS produced by BY-2 cells after PsJN treatment was lower than that produced by BY-2 cells after BR1R-2 treatment (Fig. 3). These results suggest that this system is useful as a general assay for screening bacteria for the plant immunity–activating potential.

We also confirmed that these strains (with the exception of *Bacillus* sp. BR2S-4) enhanced ROS production in *Arabidopsis* T87 cells triggered by the plant immune response elicitor flg22, a 22–amino acid peptide derived from flagellin that is known to induce ROS production^28^ (Figs. S5 and S6). These 7 strains were selected as candidate microorganisms for priming plant immune responses and then subjected to the second screening using whole plants.

### Biocontrol activity of selected microorganisms

**Figure 4:**
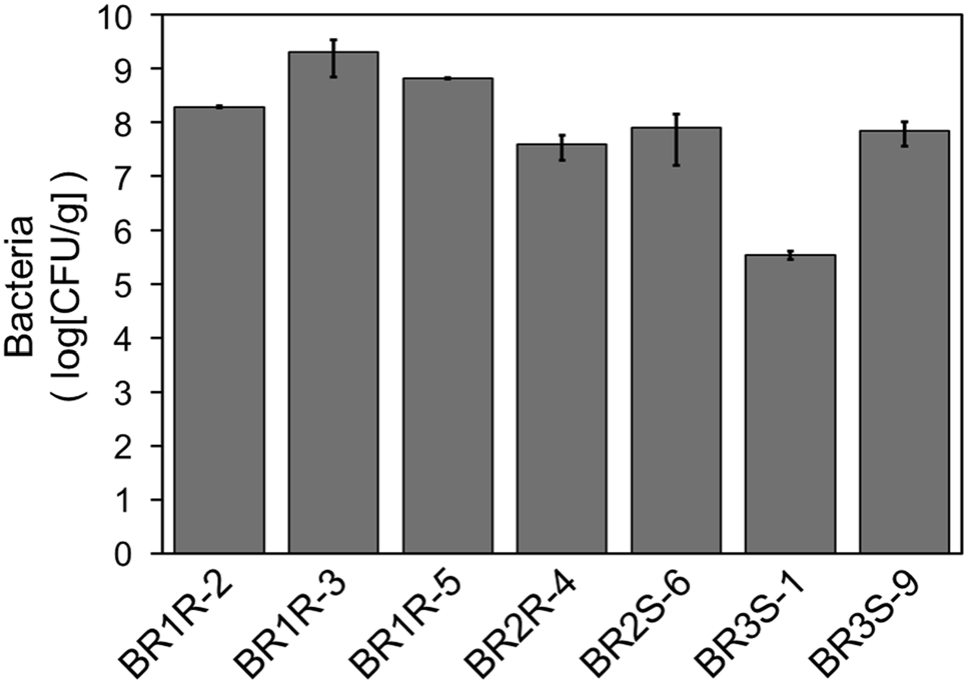
Colonization of the selected bacteria in *Arabidopsis*. Plants were inoculated with each strain of selected bacteria by immersing the root tip of 7-day-old seedlings in the bacterial cell culture solution, followed by cultivation for 7 days. After plating extracts of surface-sterilized plants on medium, colonies formed on the plate were counted. No colonies were formed for plants that received mock treatment (only the medium) instead of the bacterial cell culture solution. Average values ± SE from three independent experiments are presented.

We examined the ability of the selected bacteria to enhance disease resistance using whole *Arabidopsis* plants. Plants were inoculated with each strain of selected bacteria by immersing the root tip of 7-day-old seedlings in the bacterial cell culture solution. After cultivation for an additional 7 days, we observed that plants inoculated with each of the bacterial strains were able to grow (Figs. S7 and S8). Plating extracts of surface-sterilized bacteria-inoculated plants on NBRC802 or ISP2 agar medium revealed that the bacteria colonized the interior of the *Arabidopsis* plants (Fig. 4). The number of bacteria ranged from 10^5^ to 10^9^ colony forming units (CFU) per gram of *Arabidopsis*, depending on the bacterial strain. Inoculation with *Delftia* sp. BR1R-2 or *Arthrobacter* sp. BR2S-6 did not affect plant growth, but inoculation with the other 5 strains resulted in a significant reduction in plant growth (Figs. S7 and S8). We also confirmed that strains BR1R-2 and BR2S-6 colonized the stems and leaves (Fig. S9), indicating that these strains spread from the roots to the aerial tissues of *Arabidopsis* as endophytes.

**Figure 5:**
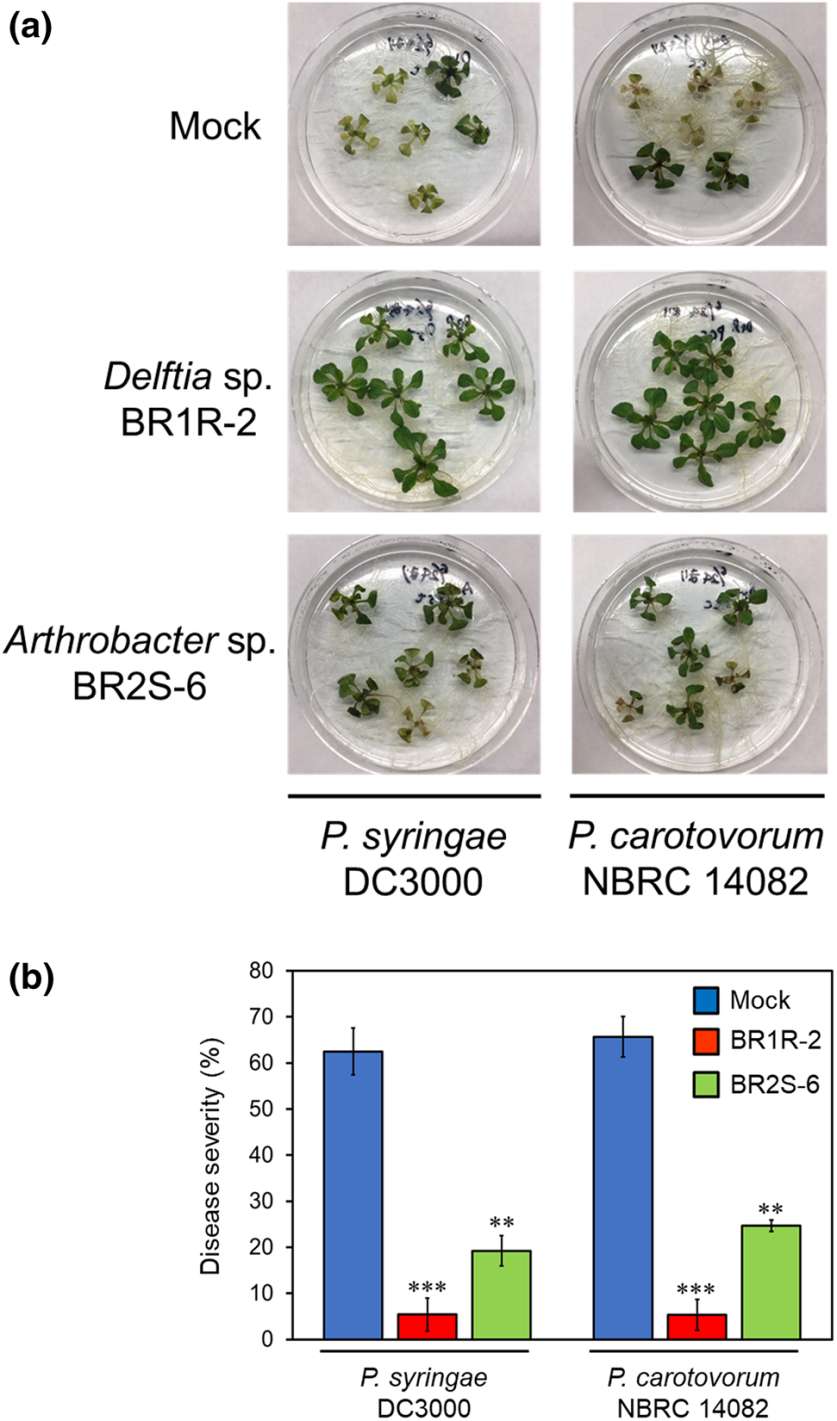
Enhancement of pathogen resistance of *Arabidopsis* by pretreatment with strains BR1R-2 and BR2S-6. BR1R-2–, BR2S-6–, or mock (only the medium)–treated *Arabidopsis* seedlings were cultivated for 7 days, and the plants were then challenged with *P. syringae* pv*. tomato* DC3000 or *P. carotovorum* subsp. *carotovorum* NBRC 14082 and cultivated for 3 days. (**a**), representative photographs; (**b**), disease severity. Disease severity is the percentage calculated by dividing the number of the damaged leaves by the number of all the leaves. Average values ± SE from three independent experiments are presented. Asterisks indicate a significant difference from the mock control based on Student’s t-test (**, P < 0.01; ***, P < 0.001).

The promising endophytes *Delftia* sp. BR1R-2 and *Arthrobacter* sp. BR2S-6 were then tested for their ability to enhance disease resistance. *Pseudomonas syringae* pv*. tomato* DC3000 and *Pectobacterium carotovorum* subsp. *carotovorum* NBRC 14082 were used as hemibiotrophic and necrotrophic bacterial pathogens, respectively. *Arabidopsis* seedlings treated with each endophyte were cultivated for 7 days, and the plants were then challenged with *P. syringae* pv*. tomato* DC3000. After cultivation for an additional 3 days, we observed that mock-treated plants exhibited severe disease symptoms of chlorosis (Fig. 5). In contrast, plants treated with strains BR1R-2 and BR2S-6 exhibited significantly less-severe disease symptoms compared with mock-treated plants (Fig. 5). We also found that the density of strain DC3000 in *Arabidopsis* decreased to 0.9% and 7.4% in plants treated with strains BR1R-2 and BR2S-6, respectively, compared with mock-treated plants (Fig. S10). Similarly, although plants challenged with *P. carotovorum* subsp. *carotovorum* NBRC 14082 exhibited soft rot, pretreatment with strains BR1R-2 and BR2S-6 enhanced the disease resistance of *Arabidopsis* plants (Fig. 5). These results confirm that the microorganisms selected using the present screening system enhance the resistance of *Arabidopsis* plants to two different specific pathogens. The biocontrol effects of strain BR1R-2 were more pronounced than those of strain BR2S-6 under the experimental conditions used in this study (Fig. 5), and therefore was further validated.

### Effects of colonization by *Delftia* sp. BR1R-2 on the expression of defense-related genes

**Figure 6:**
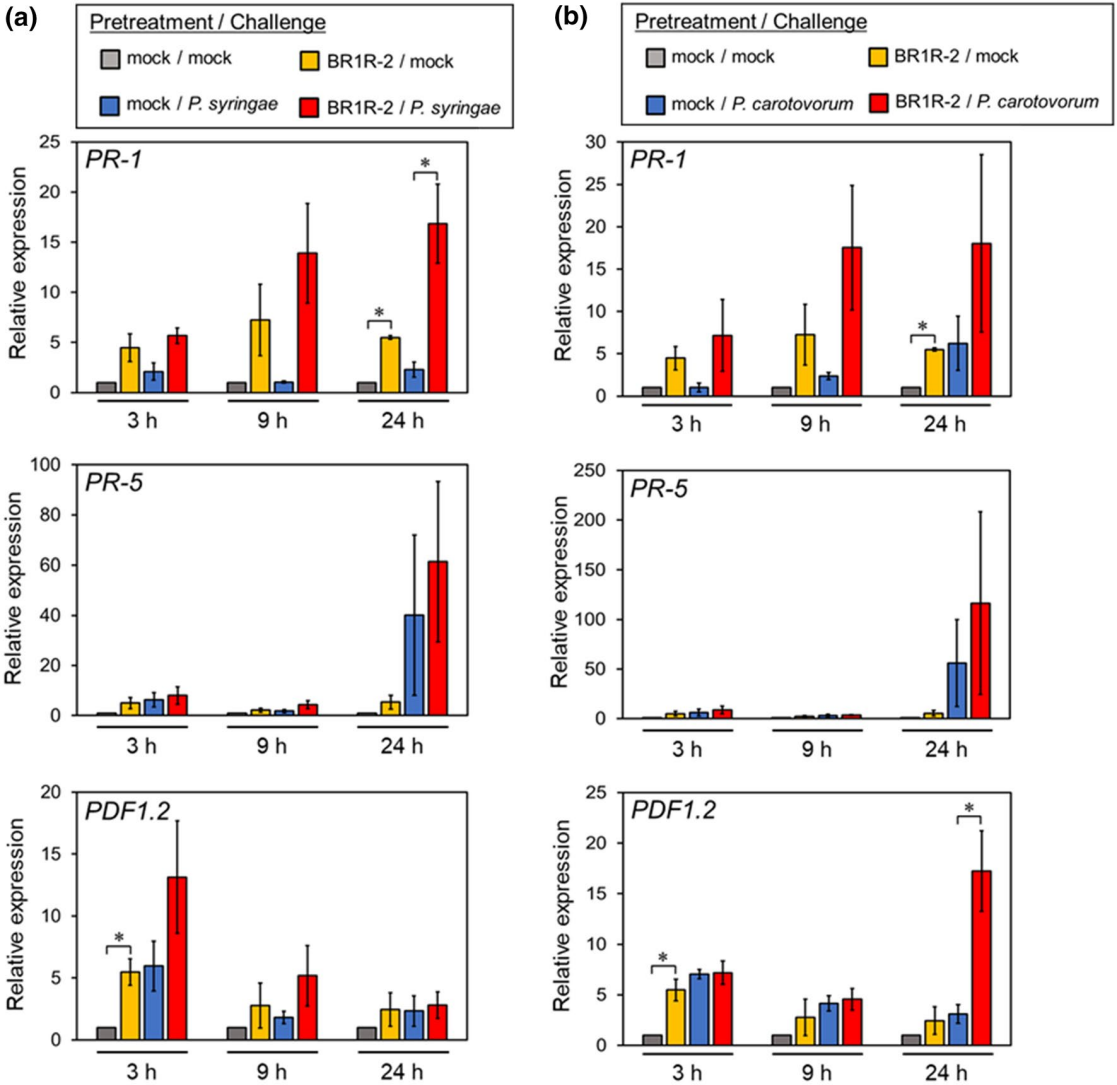
Fold-increase in *PR-1*, *PR-5*, and *PDF1.2* transcripts in *Arabidopsis* induced by pretreatment with strain BR1R-2 and pathogen challenge. BR1R-2–treated *A. thaliana* seedlings were cultivated for 7 days, and the plants were then challenged with *P. syringae* pv*. tomato* DC3000 or *P. carotovorum* subsp. *carotovorum* NBRC 14082 and cultivated for 3 days. *Arabidopsis* plants were pretreated with strain BR1R-2 (yellow bar) or mock treatment (only the medium, gray bar) and challenged with mock inoculum (only sterile water containing 0.025% Silwet L-77) instead of pathogen. In another experiment, *Arabidopsis* plants were pretreated with strain BR1R-2 (red bar) or mock treatment (only the medium, blue bar) and challenged with pathogen. (**a**), challenge with strain DC3000; (**b**), challenge with strain NBRC 14082. Average values ± SE from three independent experiments are presented. Asterisks indicate a significant difference from the mock control based on Student’s *t*-test (*, *P* < 0.05).

In order to examine the mechanism by which *Delftia* sp. BR1R-2 enhances disease resistance in *Arabidopsis*, the expression patterns of various defense-related genes (*PR-1*, *PR-5*, and *PDF1.2*) in the aerial tissues of *Arabidopsis* plants were analyzed using reverse transcription–quantitative polymerase chain reaction (RT-qPCR). Activation of defense responses via the SA signaling pathway is accompanied by expression of *PR-1* and *PR-5*, whereas *PDF1.2* is a marker of the JA/ET signaling pathway^29,30^. We first evaluated the effects of BR1R-2 colonization on gene expression (Fig. 6, gray and yellow bars). RT-qPCR analysis revealed that colonization by strain BR1R-2 induced the expression of *PR-1*, *PR-5*, and *PDF1.2*, although the expression of *PR-5* was induced at a lower level than the other two genes. These results suggest that strain BR1R-2 simultaneously activates both the SA and JA/ET signaling pathways in *Arabidopsis*.

We then evaluated the effects of BR1R-2 colonization on pathogen-induced gene expression in *Arabidopsis*. Plants grown from BR1R-2– and mock-treated seedlings were challenged with *P. syringae* pv*. tomato* DC3000 (Fig. 6a, blue and red bars). BR1R-2–treated plants exhibited higher expression of *PR-1* compared with mock-treated plants at 9 and 24 h after infection. The expression of *PR-5* was higher in BR1R-2–treated plants than mock-treated plants at 24 h after infection, whereas *PDF1.2* expression in BR1R-2–treated plants was upregulated at 3 h post-infection. Similarly, the expression of *PR-1*, *PR-5*, and *PDF1.2* induced by *P. carotovorum* subsp. *carotovorum* NBRC 14082 in *Arabidopsis* was enhanced by pretreatment with strain BR1R-2 (Fig. 6b, blue and red bars). The expression of *PDF1.2* was upregulated at 24 h after infection with strain NBRC 14082, whereas its expression was high at 3 h after infection with strain DC3000. These results indicate that strain BR1R-2 primes and potentiates the expression of plant defense-related genes induced by pathogen infection.

## Discussion

Assuming that pesticide-free vegetable plants can be viable as a result of colonization by beneficial microorganisms, we isolated bacteria from *B. rapa* var. *perviridis* grown by organic farming without the use of pesticides. A total of 31 bacterial strains were recovered from the interior of the plant, and these strains belonged to 3 phyla, *Firmicutes*, *Actinobacteria*, and *Proteobacteria*. It has been reported that bacterial endophytes are typically dominated by 4 phyla, *Proteobacteria*, *Actinobacteria*, *Firmicutes*, and *Bacteroidetes*^31,32^. The present results indicate that the bacterial strains isolated from *B. rapa* var. *perviridis* belong to the same groups at the phylum level as bacteria isolated from other plant species. Although endophytes that colonize *Brassicaceae* plants such as *A. thaliana*, *B. napus*, and *B. campestris* have been studied^33,34^, to our knowledge, this is the first report describing the diversity of bacteria isolated from the interior parts of *B. rapa* var. *perviridis*. We isolated 9 *Bacillus*, 5 *Pseudomonas*, and 2 *Stenotrophomonas* strains. Bacteria of these genera have frequently been recovered from *Brassicaceae* plants. In addition, we isolated 2 *Brevibacterium*, 3 *Glutamicibacter*, 3 *Arthrobacter*, and 2 *Paenarthrobacter* strains, as well as 1 *Delftia* strain. Interestingly, few reports have described bacteria of these genera in the microbiome of *Brassicaceae*. The number of bacterial strains isolated from roots and leaves were relatively small. This might be attributed to harsh conditions used for the surface sterilization in this study.

In this study, we developed a novel system for screening for microorganisms that activate plant immune responses based on plant–microbe interactions using cultured plant cells. The bacteria isolated from the interior of *B. rapa* var. *perviridis* plants were examined using the screening system with cryptogein-induced ROS production in tobacco BY-2 cells as a marker. A total of 8 bacterial strains were selected using this screening system (Figs. 3 and S3b). Interestingly, although 4 of these 8 strains (BR1R-3, BR2S-4, BR3S-1, and BR3S-9) did not induce BY-2 cells to produce ROS in the absence of cryptogein (Fig. S1), the 4 strains did enhance cryptogein-induced ROS production in the cultured plant cells. We also found that 7 of the 8 bacterial strains enhanced flg22-induced ROS production in *Arabidopsis* T87 cells (Fig. S6). Thus, using this screening system, bacteria belonging to a variety of genera within 3 phyla were selected as candidate microorganisms for priming plant immune responses. In this study, we subjected bacteria after 24-h cultivation to the assays in order to rapidly screen many bacterial strains. On the other hand, since cultivation time affects the growth phase, detailed examination of the time would be needed to optimize plant immunity–activating potential of each strain. It should also be noted that the developed method cannot select microorganisms that activate plant immune responses without enhancement of elicitor-induced ROS production.

Endophytes are generally preferable as biocontrol agents due to their inherent ability to stably colonize in the interior of plants. Characteristics such as motility, adhesion, and cell-wall degradation activity are reportedly required for such colonization^35,36^. We confirmed that 7 bacterial strains selected using the proposed screening system were capable of colonizing the interior of *Arabidopsis* plants (Fig. 4). The number of bacteria colonizing plants was relatively high (Fig. 4). The medium used here contained 10 g/l sucrose as a carbon source for the plant, which is likely a good carbon source for the bacteria as well. However, 5 of these 7 strains caused a significant reduction in plant growth (Figs. S7 and S8). This growth inhibition was not correlated with the number of bacteria colonizing the plants (Figs. 4 and S8). One possible explanation is that these strains induce defense responses too strongly. Strong induction of defense responses in plants is often accompanied by cell cycle arrest or growth inhibition^21,37,38^. In contrast, 2 of the 7 bacterial strains, *Delftia* sp. BR1R-2 and *Arthrobacter* sp. BR2S-6, colonized the interior of *Arabidopsis* plants without inhibiting their growth (Figs. S7 and S8). These two endophytes endowed *Arabidopsis* with resistance to both hemibiotrophic and necrotrophic bacterial pathogens (Fig. 5). Therefore, strains BR1R-2 and BR2S-6 could be useful biocontrol agents. Strain BR1R-2 is the first bacterium of the genus *Delftia* shown to function as a biocontrol agent and exhibited more pronounced biocontrol effects on *Arabidopsis* than strain BR2S-6 (Fig. 5).

*Delftia* sp. BR1R-2 was further examined in order to elucidate the mechanism by which it enhances pathogen resistance in *Arabidopsis*. Nonpathogenic bacteria reportedly enhance disease resistance by stimulating plant defense-related genes, as described above. Here, we investigated the expression of *PR-1* and *PR-5*, which are generally involved in the SA signaling pathway, and the expression of *PDF1.2*, which is involved in the JA/ET signaling pathway. Colonization by strain BR1R-2 induced the expression of all three genes (Fig. 6). These results suggest that strain BR1R-2 simultaneously activates the SA and JA/ET signaling pathways in *Arabidopsis* and that the resulting expression of defense-related genes provides resistance to two different pathogens. The biocontrol activity of most nonpathogenic bacteria involves stimulation of either pathway (primarily the JA/ET signaling pathway), whereas the number of bacteria that activate both pathways is limited^39^. For example, defense responses mediated by the rhizobacterium *Bacillus cereus* AR156 are dependent on both pathways^39^. Furthermore, the expression of *PR-1*, *PR-5*, and *PDF1.2* induced by the pathogens in the present study was enhanced by pretreatment with strain BR1R-2 (Fig. 6). These results indicate that strain BR1R-2 enhances the pathogen resistance of *Arabidopsis* by priming its immune responses.

**Figure 7:**
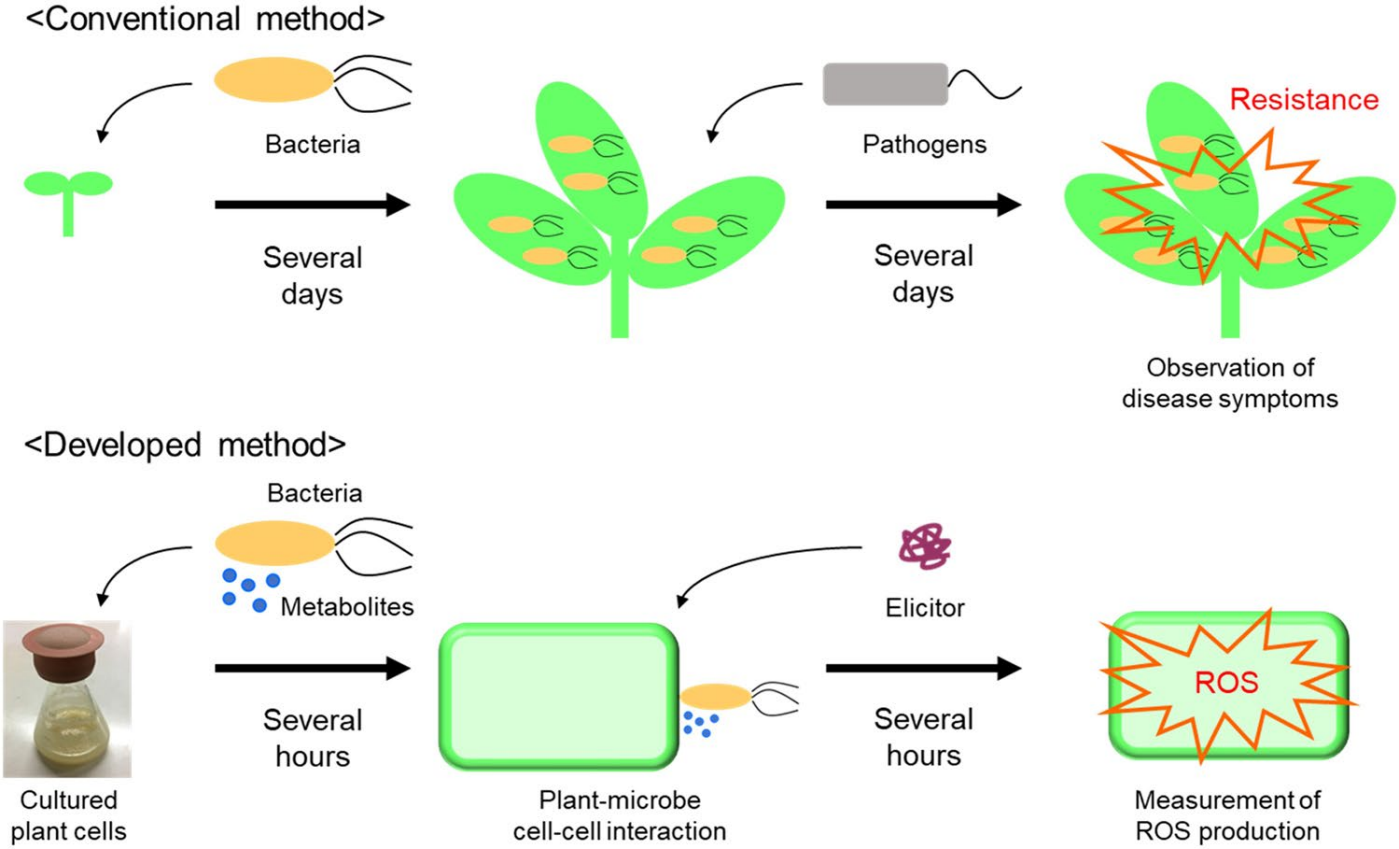
Schematic illustration of the developed method compared to the conventional method.

In conclusion, we described a general strategy for exploring the potential of microorganisms to activate plant immune responses based on plant–microbe interactions using cultured plant cells. The value of this strategy was demonstrated by identifying novel plant immunity–activating bacteria, *Delftia* sp. BR1R-2 and *Arthrobacter* sp. BR2S-6. The developed method using cultured plant cells enables rapid direct screening of microorganisms for plant immunity–activating potential, thus reducing the number of samples subjected to laborious assays using whole plants (Fig. 7). Therefore, this approach should be readily applicable to large-scale screening for plant immunity–activating microorganisms from a variety of environments.

## Materials and methods

### Isolation and identification of bacteria from the interior of *B. rapa* var. *perviridis*

*Brassica rapa* var. *perviridis* plants were grown by organic farming without the use of pesticides at the Suzuki Farm (Tachikawa, Tokyo, Japan) and collected between May and July 2017. The plants were separated into roots, stems, and leaves. The plant tissues were then washed with running tap water and aseptically sectioned into 1-cm fragments. These fragments were surface-sterilized by dipping in 5% sodium hypochlorite for 3 min, followed by 70% ethanol for 2 min, after which they were rinsed with sterile water for a few minutes, according to a previous report^24^, with some modifications. Each fragment was further cut and placed onto NBRC802 or ISP2 agar medium and incubated at 30 °C for approximately 1 month. The final rinse water was also plated onto each medium to confirm the effectiveness of the surface sterilization. After incubation, single-colony isolation was repeated for colonies formed around the tissues. NBRC802 medium contained (per liter) Hipolypepton (10 g), Bacto yeast extract (2 g), and MgSO_4_·7H_2_O (1 g) (pH 7.0). ISP2 medium contained (per liter) Bacto yeast extract (4 g), Bacto malt extract (10 g), and glucose (4 g) (pH 7.3).

Taxonomic identification of isolated bacteria was performed based on the 16S rDNA sequence. The DNA was amplified from colonies by polymerase chain reaction (PCR) using two oligonucleotide primers, 9F 5′-GAGTTTGATCCTGGCTCAG-3′ and 1541R 5′-AAGGAGGTGATCCAGCC-3′. PCR was performed using KOD FX Neo polymerase (Toyobo, Osaka, Japan) according to the manufacturer’s recommendations under the following conditions: 94 °C for 2 min, followed by 40 cycles of 98 °C for 10 s, 68 °C for 2 min, and 72 °C for 10 min. After purification, the amplified DNAs were sequenced by Eurofins (Tokyo, Japan). The sequences of the 5′-terminal region (ca. 500 bp) were determined for all strains except BR2R-1, for which the 3′-terminal region (ca. 500 bp) sequence was determined because the 16S rDNA sequence contains an insertion (ca. 300 bp) in the 5′-terminal region. The sequences were compared to those in the GenBank database using BLASTN (https://blast.ncbi.nlm.nih.gov/Blast.cgi). MEGA software (https://www.megasoftware.net/) was used to align the sequences and construct a neighbor-joining phylogenetic tree.

*P. phytofirmans* PsJN (DSM 17,436) was purchased from German Collection of Microorganisms and Cell Cultures. Strain PsJN was cultivated in trypticase soy broth (BD, Sparks, MD, USA) (pH 7.3) at 30 °C for 24 h.

### Plant materials and growth conditions

Suspensions of tobacco BY-2 (*Nicotiana tabacum* L. cv. Bright Yellow 2) cells were maintained by weekly dilution (1/100) with fresh Linsmaier and Skoog (LS) medium, modified according to previous reports^18,19^. The cells were maintained in the dark at 28 °C with aeration (shaking at 120 rpm). Suspensions of *A. thaliana* T87 cells were maintained by weekly dilution (2/100) with fresh Jouanneau and Péaud-Lenoël (JPL) medium^40^. The cells were maintained at 22 °C with aeration (shaking at 120 rpm) under a light intensity of 60–100 µE m^−2^ s^−1^. Although we cannot provide the plant cell lines used in our laboratory, BY-2 and T87 cells are available from RIKEN BioResource Research Center in Japan.

*Arabidopsis thaliana* Columbia-0 was employed for whole-plant experiments. Seeds were surface-sterilized by dipping in 20% sodium hypochlorite for 10 min and then washed repeatedly with sterile water. After treatment at 4 °C in the dark for 2 days, sterilized seeds were sown in 1/2 Murashige and Skoog (MS) medium (Sigma-Aldrich, St. Louis, MO, USA) supplemented with 10 g/l sucrose and solidified with 3 g/l Phytagel (Sigma-Aldrich) in Petri dishes^41,42^. The plates were then transferred to a plant growth chamber with a light intensity of 150–200 µE m^−2^ s^−1^ (16 h light/8 h dark) and temperature of 22 °C.

### Incubation of bacteria with tobacco BY-2 cells and measurement of ROS production

After cultivation in modified LS medium for 3 days, tobacco BY-2 cells were collected by centrifugation and suspended in ROS assay buffer (5 mM MES, 175 mM mannitol, 0.5 mM CaCl_2_, and 0.5 mM K_2_SO_4_ [pH 5.8]). The plant cell suspension (60 g wet cell weight/l) was incubated at room temperature on a rotary shaker (120 rpm) for 3 h. Cells of each isolated bacterial strain were cultivated in liquid NBRC802 or ISP2 medium at 30 °C for 24 h and then added to the plant cell suspension. In this process, the bacterial cell culture solution was adjusted to an optical density at 600 nm (OD_600_) of 0.8 using NBRC802 or ISP2 medium, and the solution was further diluted by a factor of 2 using ROS assay buffer. Then, 0.1 mL of the diluted solution (cells and extracellular components in a mixture of the medium and the buffer at a ratio of 50:50) was mixed with 1.9 mL of the plant cell suspension (60 g wet cell weight/l) in a well (3 mL) of a 6-well plate. In this experimental system, both cells and extracellular components produced by cells were subjected to the assays to evaluate plant–microbe interactions based on physical and chemical signals. After addition of the diluted solution of bacterial cell culture, the mixture was incubated at room temperature on a rotary shaker (120 rpm), and production of ROS was monitored using a chemiluminescence assay with luminol. The mixture was filtered, and the filtrate (10 μL) was added to Tris–HCl buffer (50 mM [pH 8.0], 150 µL), followed by the addition of luminol (Wako, Osaka, Japan; 1 mM, 25 μl) and potassium ferricyanide (6 mM, 25 µL). ROS-associated chemiluminescence was measured for 15 s using a luminometer (Centro LB 960, Berthold, Germany). Chemiluminescence was integrated and expressed as relative intensity^18,19^. Samples that exhibited relative chemiluminescence intensity more than twice as high as mock treatment were selected as positives (Fig. S1).

### Measurement of cryptogein-induced ROS production in BY-2 cells after co-incubation with bacteria

After cultivation in modified LS medium for 3 days, tobacco BY-2 cells were collected by centrifugation and suspended in ROS assay buffer. The bacterial cell culture solution was adjusted to OD_600_ of 0.8 using NBRC802 or ISP2 medium (trypticase soy broth was used for strain PsJN), and the solution was further diluted by a factor of 10 using ROS assay buffer. Then, 0.1 mL of the diluted solution (cells and extracellular components in a mixture of the medium and the buffer at a ratio of 10:90) was added to the plant cell suspension (60 g wet cell weight/l, 1.8 mL) (Fig. S2). After co-incubation at room temperature on a rotary shaker (120 rpm) for 4 h, the cells were collected by centrifugation (1000 rpm, 3 min) and suspended in fresh buffer to remove ROS scavengers and other bacteria-derived metabolites. Cryptogein (6 µM, 0.1 mL), as a plant immune response elicitor, was then added to the solution. The mixture was incubated at room temperature on a rotary shaker (120 rpm), and production of ROS was monitored using a chemiluminescence assay with luminol, as described above. Samples that exhibited relative chemiluminescence intensity more than twice as high as mock treatment were selected as positives (Fig. S3).

### Measurement of flg22-induced ROS production in T87 cells after co-incubation

*Arabidopsis* T87 cells were cultivated in JPL medium for 3 days and then collected by centrifugation and suspended in ROS assay buffer. The plant cells were co-incubated with each strain of isolated bacteria as described for tobacco BY-2 cells. The plant immune response elicitor flg22 (final concentration, 1 µM) was then added to the buffer instead of cryptogein (Fig. S5). The luminol derivative L-012 (Wako; final concentration, 50 µM) was added to the buffer simultaneously. The mixture was incubated at room temperature on a rotary shaker (120 rpm), and ROS-associated chemiluminescence was measured for 0.5 s using a luminometer.

### Treatment of whole *Arabidopsis* plants with isolated bacteria

Whole *Arabidopsis* Col-0 plants were inoculated with each strain of isolated bacteria by immersing the root tip of 7-day-old seedlings in the diluted solution of bacterial cell culture (OD_600_, 0.002) for 1 s. This solution was prepared by diluting the bacterial cell culture solution after cultivation for 24 h using NBRC802 or ISP2 medium beforehand. After inoculation, the plants were transferred to fresh 1/2 MS agar medium and further cultivated on the plate at 22 °C with a light intensity of 150–200 µE m^−2^ s^−1^ (16 h light/8 h dark) for 7 days.

To evaluate internal colonization of the plants by the isolated bacteria, inoculated plants were surface-sterilized by dipping in 5% H_2_O_2_ for 2 min^41,42^. After washing three times with sterile water, a pooled sample of 6 seedlings was homogenized in 5 mL of sterile water using a mortar and pestle. Subsequently, appropriately diluted samples were plated onto NBRC802 or ISP2 agar medium. After incubation at 30 °C for a few days, colonies formed on the plates were counted, and bacterial density was expressed as CFU per gram of plant fresh weight.

### Evaluation of resistance of *Arabidopsis* to hemibiotrophic and necrotrophic bacterial pathogens

*P. syringae* pv*. tomato* DC3000 and *P. carotovorum* subsp. *carotovorum* NBRC 14082 were used as bacterial strains pathogenic to *Arabidopsis*^42,43^. Strain DC3000 was cultivated on mannitol-glutamate (MG) agar medium containing rifampicin (50 µg mL^−1^) at 28 °C for 24 h. Strain NBRC 14082 was cultivated on NBRC802 medium at 30 °C for 24 h. MG medium contained (per liter) mannitol (10 g), L-glutamic acid (2 g), KH_2_PO_4_ (0.5 g), NaCl (0.2 g), and MgSO_4_·7H_2_O (0.2 g) (pH 7.0).

Pathogenic bacterial cell suspension (4 × 10^5^ CFU mL^−1^; 40 mL) prepared in sterile water containing 0.025% Silwet L-77 (Biomedical Science, Tokyo, Japan) was dispensed into 1/2 MS agar medium containing 14-day-old *Arabidopsis* seedlings, and the plates were incubated at room temperature for 2 min^41,42^. After the pathogen cell suspension was removed by decantation, the seedlings on the plates were rinsed twice with sterile water. The plates were then sealed with 3 M Micropore 2.5-cm surgical tape (3 M, St. Paul, MN, USA) and incubated at 22 °C with a light intensity of 150–200 µE m^−2^ s^−1^ (16 h light/8 h dark). Symptom development was observed at 3 days after infection.

To determine the growth of strain DC3000 in *Arabidopsis*, the aerial tissues of infected plants were sampled. The tissues were surface-sterilized by dipping in 5% H_2_O_2_ for 2 min^41,42^. After washing twice with sterile water, a pooled sample of 5 seedlings was homogenized in 5 mL of sterile water using a mortar and pestle. Subsequently, appropriately diluted samples were plated onto MG agar medium containing rifampicin. After incubation at 28 °C for 2 days, colonies formed on the plates were counted, and bacterial density was expressed as CFU per gram of plant fresh weight.

### Gene expression analysis

The aerial tissues of *Arabidopsis* plants were sampled at 3, 9, and 24 h after pathogen infection and ground in liquid nitrogen using a mortar and pestle. Total RNA was isolated using an RNA extraction kit (NucleoSpin RNA Plus, Takara Bio, Shiga, Japan). Reverse transcription was performed using reverse transcriptase (ReverTra Ace qPCR RT Master Mix with gDNA Remover, Toyobo). The expression levels of defense-related genes were determined by quantitative PCR using Thunderbird SYBR qPCR Mix (Toyobo) and specific primer sets. The following primers were used: *EF-1α*, forward 5′-TGAGCACGCTCTTCTTGCTTTCA-3′ and reverse 5′-GGTGGTGGCATCCATCTTGTTA-3′; *PR-1*, forward 5′-GTGGGTTAGCGAGAAGGCTA-3′ and reverse 5′-ACTTTGGCACATCCGAGTCT-3′; *PR-5*, forward 5′-TCGGCGATGGAGGATTTGAA-3′ and reverse 5′-AGCCAGAGTGACGGGAGGAAC-3′; *PDF1.2*, forward 5′-TCATGGCTAAGTTTGCTTCC-3′ and reverse 5′-AATACACACGATTTAGCACC-3′. Quantitative PCR was performed using a CFX Connect real-time system (BIO-RAD, Tokyo, Japan) according to the manufacturer’s recommendations under the following conditions: 95 °C for 1 min, followed by 40 cycles of 95 °C for 15 s, 60 °C for 1 min, and 65 °C for 15 s. The specificity of the amplifications was verified by melting curve analysis of the PCR products at the end of each experiment. The relative expression level of each gene was normalized against the expression level of *EF1α*, and calculated using the ΔΔCt method^44^.

## Supplementary Information

Supplementary Information

## References

[1] Dangl JL, Jones JD (2001). Plant pathogens and integrated defence responses to infection. Nature.

[2] Jones JD, Dangl JL (2006). The plant immune system. Nature.

[3] Bigeard J, Colcombet J, Hirt H (2015). Signaling mechanisms in pattern-triggered immunity (PTI). Mol. Plant.

[4] Peng Y, van Wersch R, Zhang Y (2018). Convergent and divergent signaling in PAMP-triggered immunity and effector-triggered immunity. Mol. Plant Microbe Interact..

[5] Ramirez-Prado JS, Abulfaraj AA, Rayapuram N, Benhamed M, Hirt H (2018). Plant immunity: from signaling to epigenetic control of defense. Trends Plant Sci..

[6] Ryals J, Uknes S, Ward E (1994). Systemic acquired resistance. Plant Physiol..

[7] Lawton K (1995). Systemic acquired resistance in *Arabidopsis* requires salicylic acid but not ethylene. Mol. Plant Microbe Interact..

[8] van Loon LC, Bakker PA, Pieterse CM (1998). Systemic resistance induced by rhizosphere bacteria. Annu. Rev. Phytopathol..

[9] Pieterse CM (1998). A novel signaling pathway controlling induced systemic resistance in *Arabidopsis*. Plant Cell.

[10] van Peer R, Niemann GJ, Schippers B (1991). Induced resistance and phytoalexin accumulation in biological control of fusarium wilt of carnation by *Pseudomonas* sp. strain WCS417r. Phytopathology.

[11] Conn VM, Walker AR, Franco CM (2008). Endophytic actinobacteria induce defense pathways in *Arabidopsis thaliana*. Mol. Plant Microbe Interact..

[12] Timmermann T (2017). *Paraburkholderia phytofirmans* PsJN protects *Arabidopsis thaliana* against a virulent strain of *Pseudomonas syringae* through the activation of induced resistance. Mol. Plant Microbe Interact..

[13] Esmaeel Q (2018). *Paraburkholderia phytofirmans* PsJN-Plants Interaction: From Perception to the Induced Mechanisms. Front Microbiol..

[14] Timmusk S, Behers L, Muthoni J, Muraya A, Aronsson AC (2017). Perspectives and challenges of microbial application for crop improvement. Front. Plant Sci..

[15] Van Wees SC, Van der Ent S, Pieterse CM (2008). Plant immune responses triggered by beneficial microbes. Curr. Opin. Plant Biol..

[16] Kurusu T, Higaki T, Kuchitsu K, Gunawardena A, McCabe P (2015). Programmed cell death in plant immunity: cellular reorganization, signaling and cell cycle dependence in cultured cells as a model system. Plant programmed cell death.

[17] Nagata T, Nemoto Y, Hasegawa S (1992). Tobacco BY-2 cell line as the “HeLa” cell in the cell biology of higher plants. Int. Rev. Cytol..

[18] Kadota Y (2006). Continuous recognition of the elicitor signal for several hours is prerequisite for induction of cell death and prolonged activation of signaling events in tobacco BY-2 cells. Plant Cell Physiol..

[19] Kadota Y (2004). Cryptogein-induced initial events in tobacco BY-2 cells: pharmacological characterization of molecular relationship among cytosolic Ca^2+^ transients, anion efflux and production of reactive oxygen species. Plant Cell Physiol..

[20] Kadota K, Kuchitsu K (2006). Regulation of elicitor-induced defense responses by Ca^2+^ channels and cell cycle in tobacco BY-2 cells. Biotechnol. Agric. For..

[21] Kadota Y (2004). Crosstalk between elicitor-induced cell death and cell cycle regulation in tobacco BY-2 cells. Plant J..

[22] Kadota Y (2005). Cell-cycle dependence of elicitor-induced signal transduction in tobacco BY-2 cells. Plant Cell Physiol..

[23] Higaki T, Kurusu T, Hasezawa S, Kuchitsu K (2011). Dynamic reorganization and function of the cytoskeleton and vacuoles in defense responses and programmed cell death. J. Plant Res..

[24] Coombs JT, Franco CM (2003). Isolation and identification of actinobacteria from surface-sterilized wheat roots. Appl. Environ. Microbiol..

[25] Baz M (2012). Calcium- and ROS-mediated defence responses in BY2 tobacco cells by nonpathogenic *Streptomyces* sp. J. Appl. Microbiol..

[26] Mohamed KH (2015). Deciphering the dual effect of lipopolysaccharides from plant pathogenic *Pectobacterium*. Plant Signal Behav..

[27] Bordiec S (2011). Comparative analysis of defence responses induced by the endophytic plant growth-promoting rhizobacterium *Burkholderia phytofirmans* strain PsJN and the non-host bacterium *Pseudomonas syringae* pv. pisi in grapevine cell suspensions. J. Exp. Bot..

[28] Yi SY, Shirasu K, Moon JS, Lee SG, Kwon SY (2014). The activated SA and JA signaling pathways have an influence on flg22-triggered oxidative burst and callose deposition. PLoS ONE.

[29] Uknes S (1992). Acquired resistance in *Arabidopsis*. Plant Cell.

[30] van Loon LC, van Stein EA (1999). The families of pathogenesis-related proteins, their activities, and comparative analysis of PR-1 type proteins. Physiol. Mol. Plant Pathol..

[31] Bulgarelli D, Schlaeppi K, Spaepen S, Ver Loren van Themaat E, Schulze-Lefert P (2013). Structure and functions of the bacterial microbiota of plants. Annu. Rev. Plant Biol..

[32] Hardoim PR (2015). The hidden world within plants: ecological and evolutionary considerations for defining functioning of microbial endophytes. Microbiol. Mol. Biol. Rev..

[33] Card SD (2015). Beneficial endophytic microorganisms of *Brassica*: A review. Biol. Control.

[34] Haque MA, Yun HD, Cho KM (2016). Diversity of indigenous endophytic bacteria associated with the roots of Chinese cabbage (*Brassica campestris* L.) cultivars and their antagonism towards pathogens. J. Microbiol..

[35] Liu H (2017). Inner plant values: diversity, colonization and benefits from endophytic bacteria. Front. Microbiol..

[36] Reinhold-Hurek B, Hurek T (2011). Living inside plants: bacterial endophytes. Curr. Opin. Plant Biol..

[37] Noutoshi Y (2005). A single amino acid insertion in the WRKY domain of the arabidopsis TIR-NBS-LRR-WRKY-type disease resistance protein SLH1 (sensitive to low humidity 1) causes activation of defense responses and hypersensitive cell death. Plant J..

[38] Shirano Y, Kachroo P, Shah J, Klessig DF (2002). A gain-of-function mutation in an arabidopsis toll interleukin1 receptor–nucleotide binding site–leucine-rich repeat type R gene triggers defense responses and results in enhanced disease resistance. Plant Cell.

[39] Niu DD (2011). The plant growth-promoting rhizobacterium *Bacillus cereus* AR156 induces systemic resistance in *Arabidopsis thaliana* by simultaneously activating salicylate- and jasmonate/ethylene-dependent signaling pathways. Mol. Plant Microbe Interact..

[40] Begum P, Fugetsu B (2013). Induction of cell death by graphene in *Arabidopsis thaliana* (Columbia ecotype) T87 cell suspensions. J. Hazard. Mater..

[41] Ishiga Y, Ishiga T, Ichinose Y, Mysore KS (2017). *Pseudomonas syringae* flood-inoculation method in *Arabidopsis*. Bio-Protoc..

[42] Ishiga Y, Ishiga T, Uppalapati SR, Mysore KS (2011). *Arabidopsis* seedling flood-inoculation technique: a rapid and reliable assay for studying plant-bacterial interactions. Plant Methods.

[43] Seo ST, Furuya N, Lim CK, Takanami Y, Tsuchiya K (2003). Phenotypic and genetic characterization of Erwinia carotovora from mulberry (*Morus* spp.). Plant Pathol..

[44] Scuderi G, Polizzi G, Cirvilleri G (2011). Quantitative RT-PCR expression analysis of lipodepsipeptides synthetase and defence-related genes in orange fruit in response to antagonist–pathogen interaction. J. Phytopathol..

