# Supplementary material for: An efficient direct screening system for microorganisms that activate plant immune responses based on plant–microbe interactions using cultured plant cells
Source: Sci Rep. 2021 Apr 1;11:7396. doi: 10.1038/s41598-021-86560-0 (PMC8016971; doi:10.1038/s41598-021-86560-0)
Supplement: Supplementary file 1 — Supplementary Information [file 41598_2021_86560_MOESM1_ESM.pdf]

# Supplementary Information

# An Efficient Direct Screening System for Microorganisms that Activate Plant Immune Responses Based on Plant-Microbe Interactions Using Cultured Plant Cells

Mari Kurokawa, Masataka Nakano, Nobutaka Kitahata, Kazuyuki Kuchitsu, Toshiki Furuya

---

Department of Applied Biological Science, Faculty of Science and Technology, Tokyo University of Science, 2641, Yamazaki, Noda, Chiba 278-8510, Japan

Correspondence and requests for materials should be addressed to T.F. (email: tfuruya@rs.tus.ac.jp)

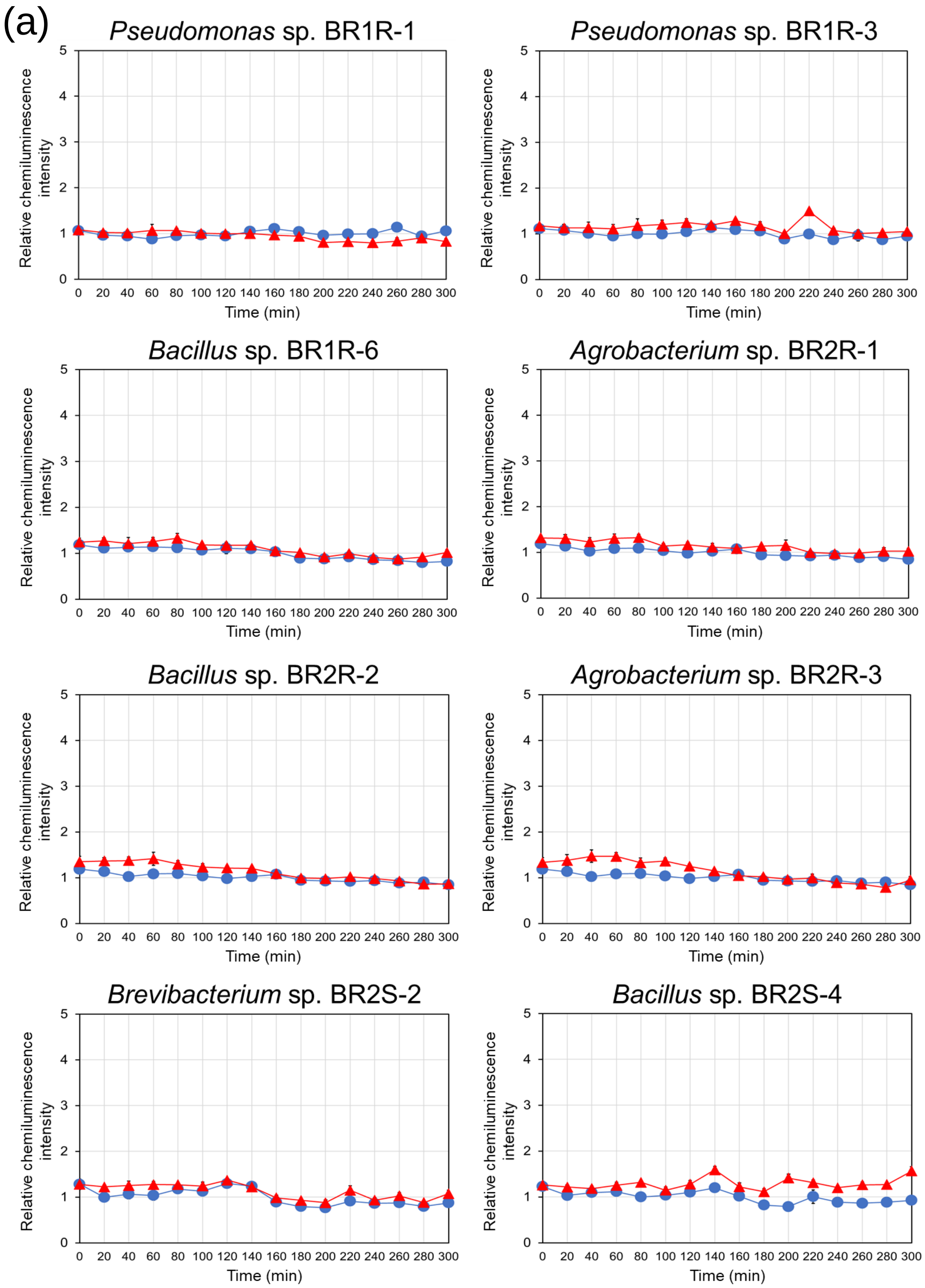
(a)
Pseudomonas sp. BR1R-1
Pseudomonas sp. BR1R-3
Bacillus sp. BR1R-6
Agrobacterium sp. BR2R-1
Bacillus sp. BR2R-2
Agrobacterium sp. BR2R-3
Brevibacterium sp. BR2S-2
Bacillus sp. BR2S-4
Relative chemiluminescence intensity
Time (min)
0 1 2 3 4 5
0 20 40 60 80 100 120 140 160 180 200 220 240 260 280 300

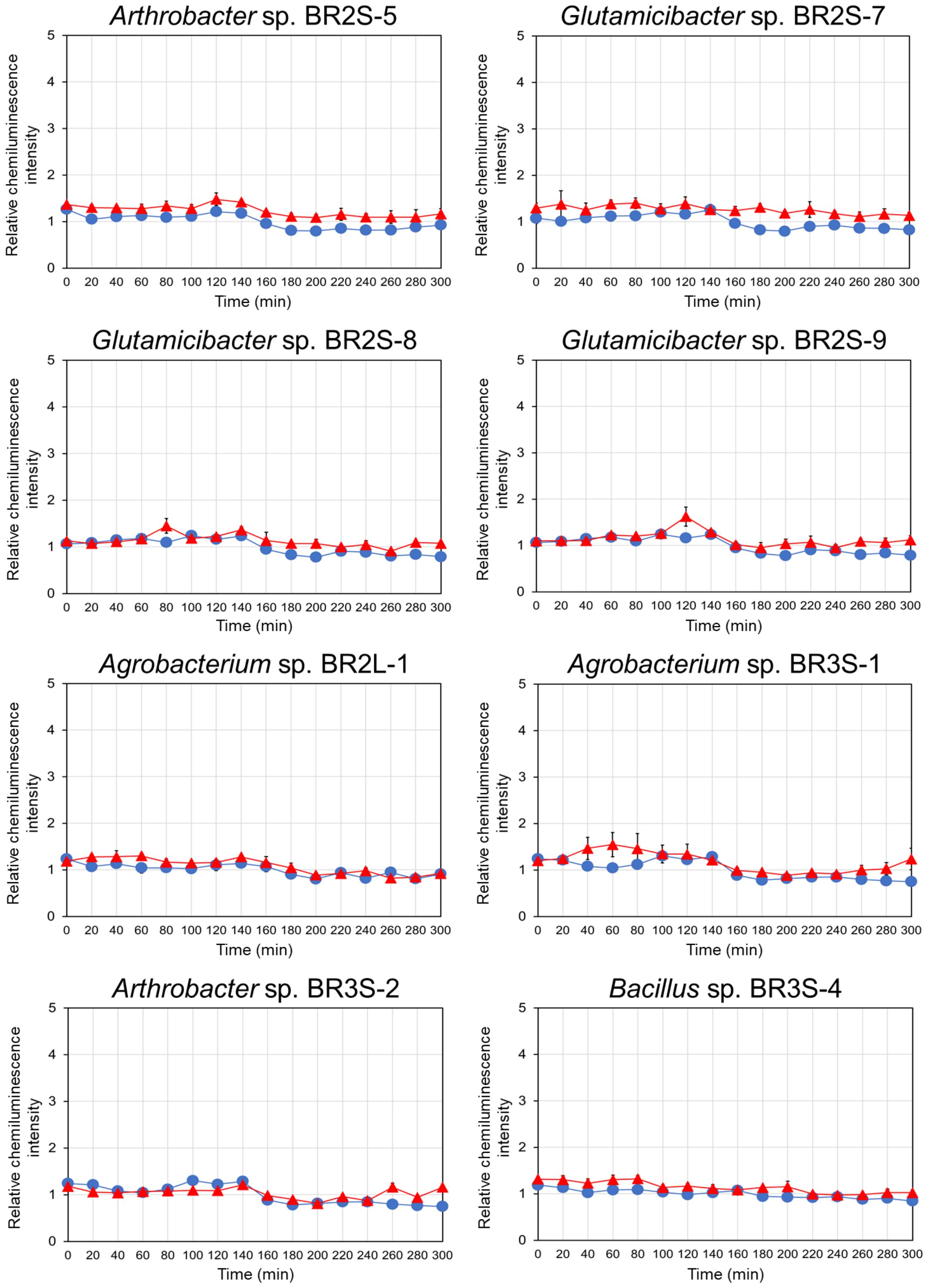
Arthrobacter sp. BR2S-5
Glutamicibacter sp. BR2S-7
Glutamicibacter sp. BR2S-8
Glutamicibacter sp. BR2S-9
Agrobacterium sp. BR2L-1
Agrobacterium sp. BR3S-1
Arthrobacter sp. BR3S-2
Bacillus sp. BR3S-4
Relative chemiluminescence intensity
Time (min)
0 1 2 3 4 5
0 20 40 60 80 100 120 140 160 180 200 220 240 260 280 300

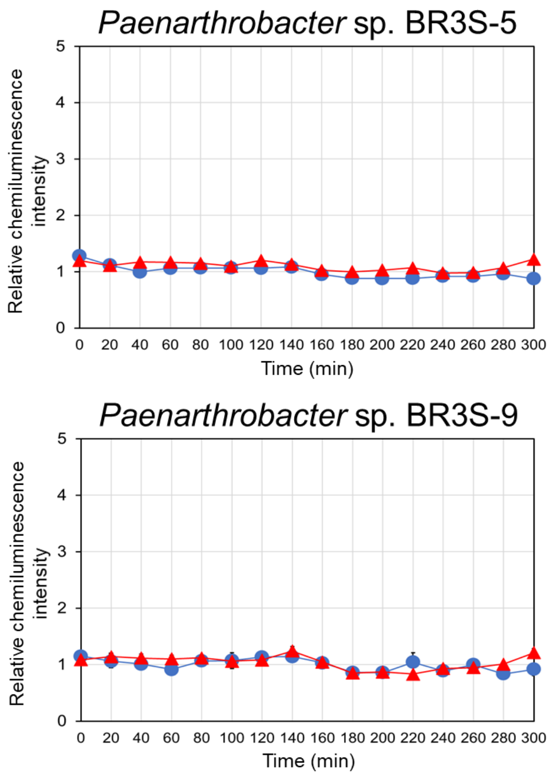
Paenarthrobacter sp. BR3S-5
Bacillus sp. BR3S-6
Paenarthrobacter sp. BR3S-9
Relative chemiluminescence intensity
Time (min)

(b)

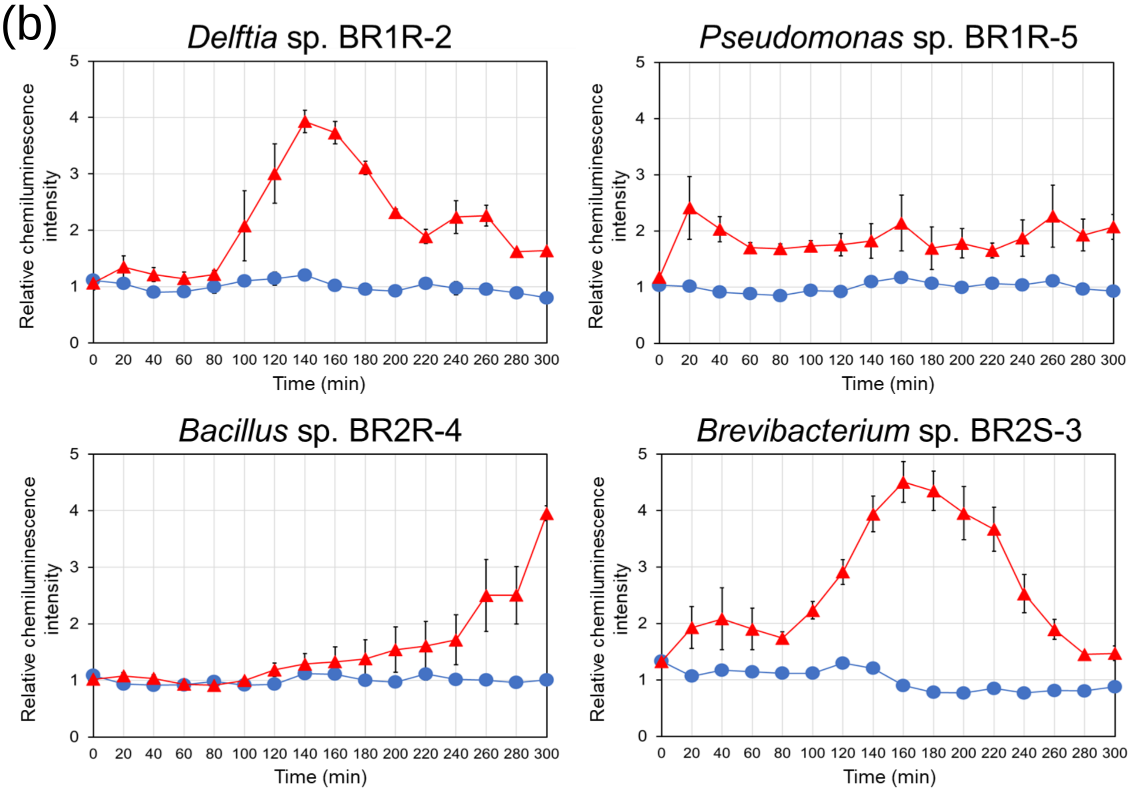
Delftia sp. BR1R-2
Pseudomonas sp. BR1R-5
Bacillus sp. BR2R-4
Brevibacterium sp. BR2S-3
Relative chemiluminescence intensity
Time (min)

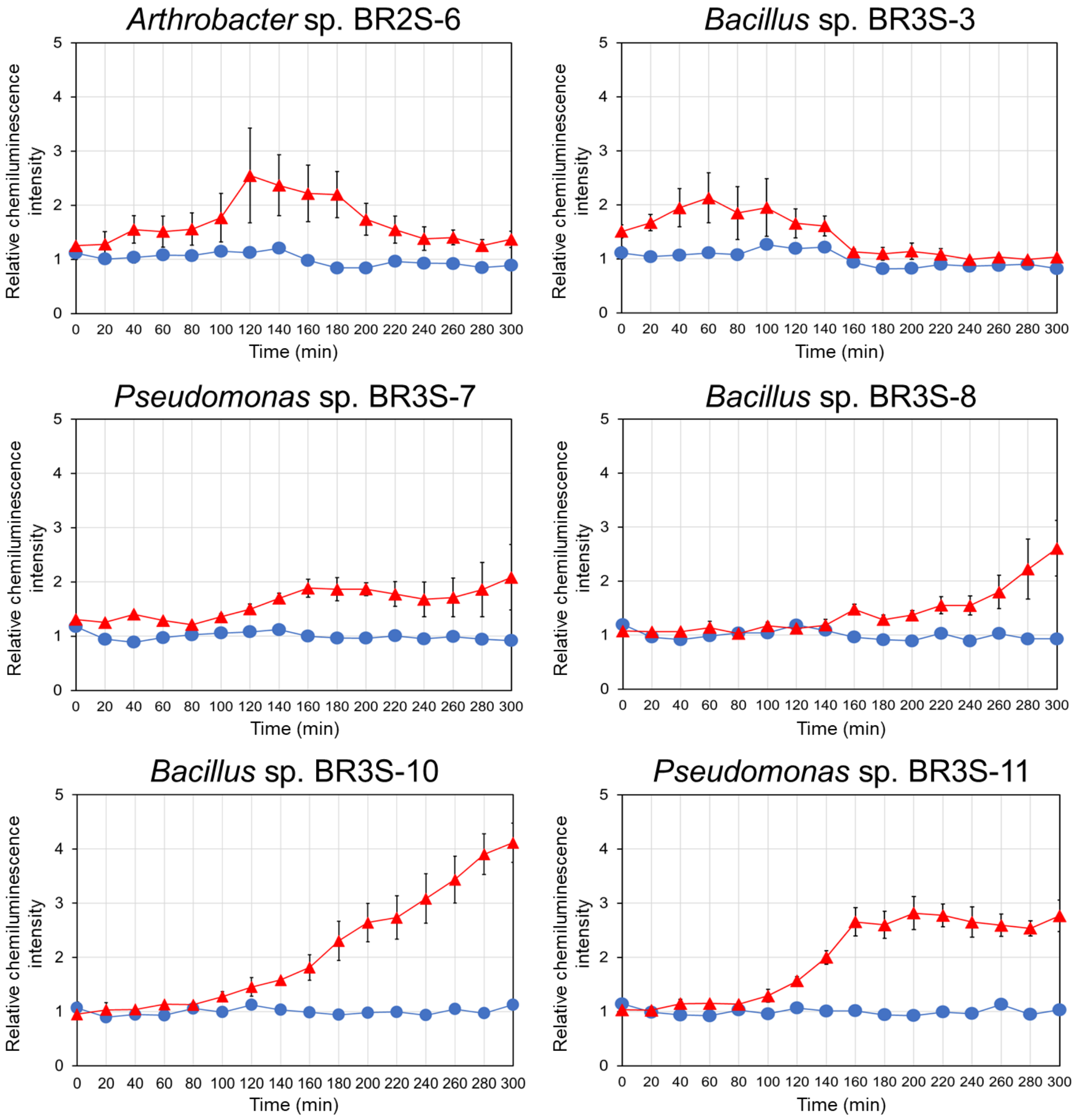


**Figure S1.** ROS production in BY-2 cells co-incubated with bacteria. BY-2 cells were co-incubated with bacteria of each isolated strain (Δ) or mock treatment (only a mixture of the medium and the buffer, ○). (a), Bacteria that did not induce ROS production; (b) Bacteria that induced ROS production. ROS production was monitored by chemiluminescence. The average value of the mock control was expressed as 1.0. Average values ± SE from three independent experiments are presented.

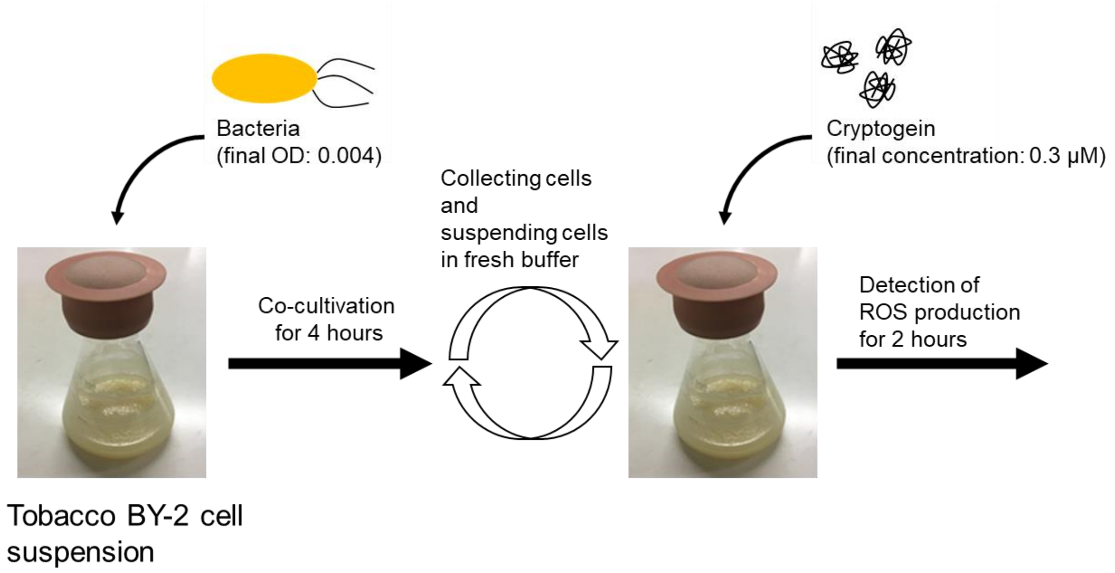


**Figure S2.** Schematic illustration of assays for cryptogein-induced ROS production in BY-2 cells co-incubated with bacteria.

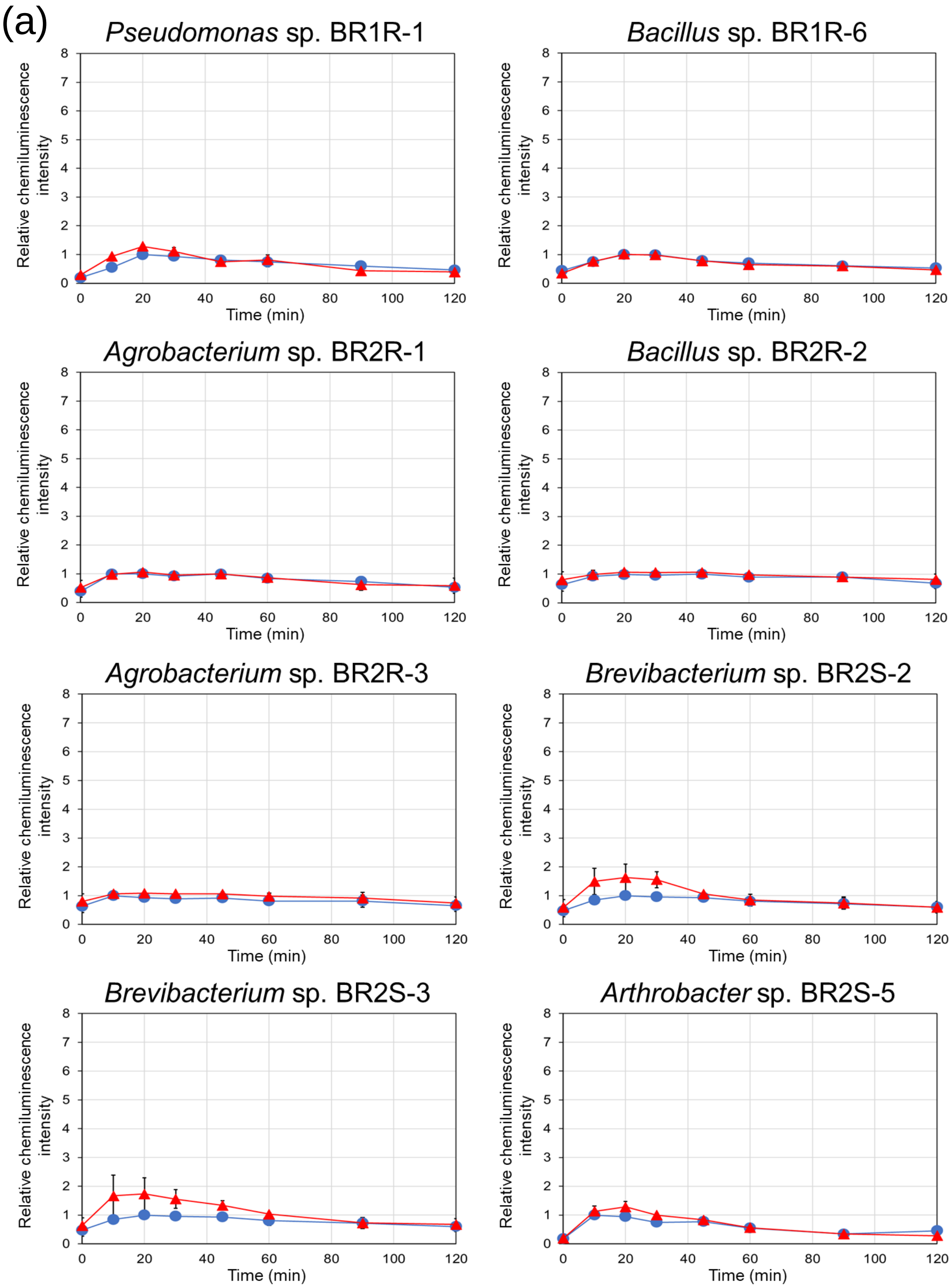
(a)
Pseudomonas sp. BR1R-1
Bacillus sp. BR1R-6
Agrobacterium sp. BR2R-1
Bacillus sp. BR2R-2
Agrobacterium sp. BR2R-3
Brevibacterium sp. BR2S-2
Brevibacterium sp. BR2S-3
Arthrobacter sp. BR2S-5
Relative chemiluminescence intensity
Time (min)

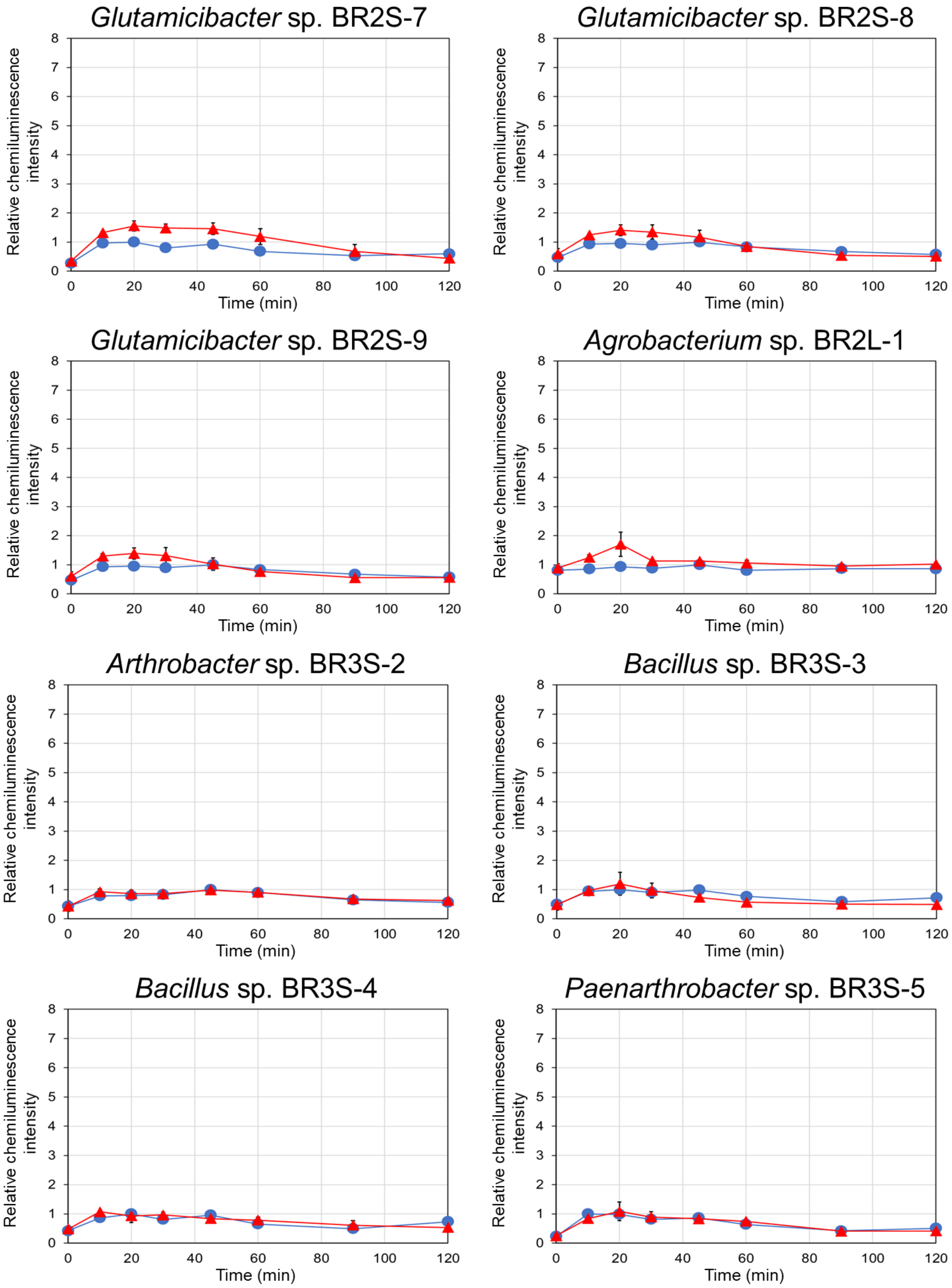

*Glutamicibacter* sp. BR2S-7
*Glutamicibacter* sp. BR2S-8
*Glutamicibacter* sp. BR2S-9
*Agrobacterium* sp. BR2L-1
*Arthrobacter* sp. BR3S-2
*Bacillus* sp. BR3S-3
*Bacillus* sp. BR3S-4
*Paenarthrobacter* sp. BR3S-5
Relative chemiluminescence intensity
Time (min)
0
1
2
3
4
5
6
7
8
20
40
60
80
100
120

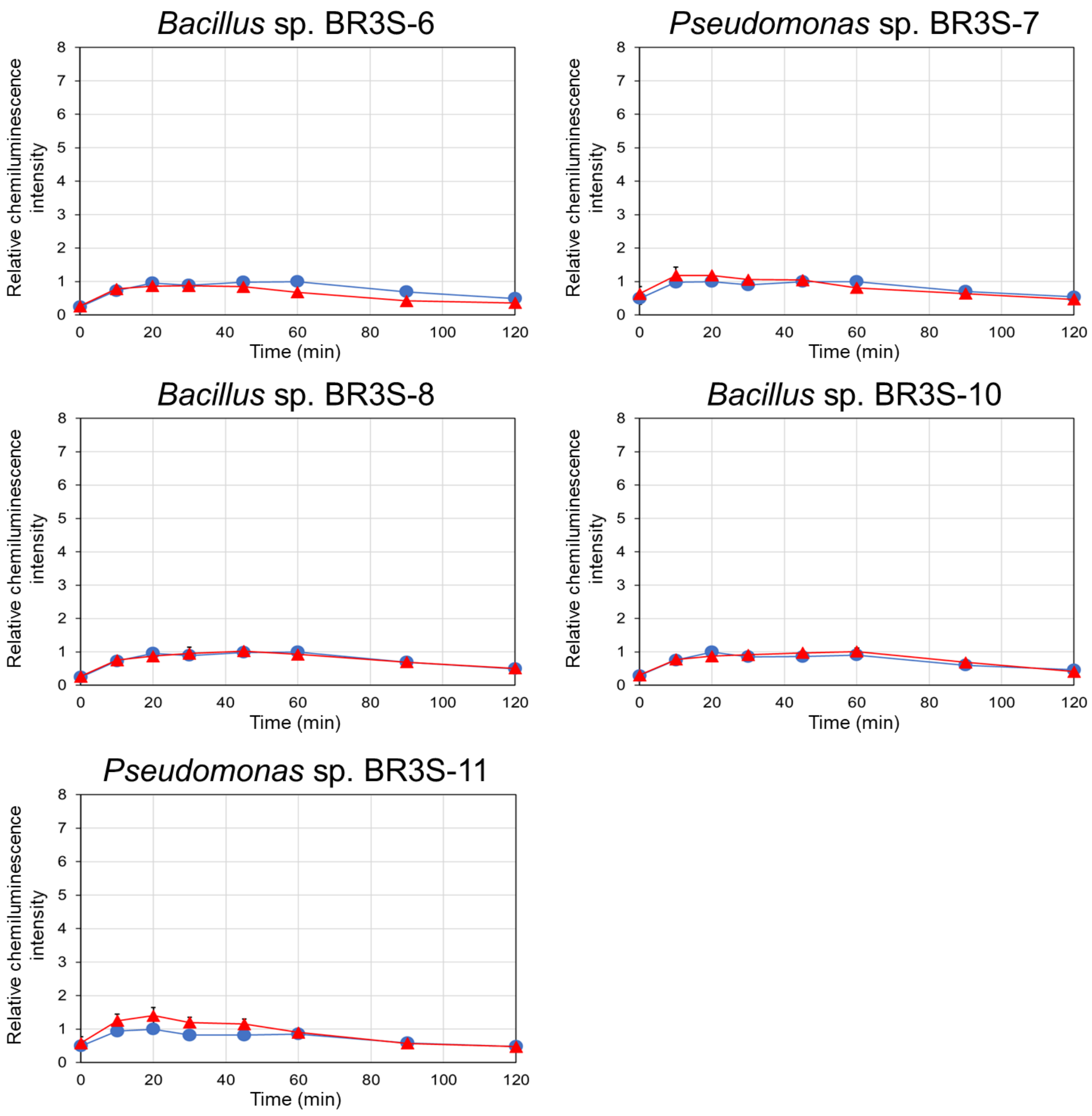
Bacillus sp. BR3S-6
Pseudomonas sp. BR3S-7
Bacillus sp. BR3S-8
Bacillus sp. BR3S-10
Pseudomonas sp. BR3S-11
Relative chemiluminescence intensity
Time (min)
0
1
2
3
4
5
6
7
8
0
20
40
60
80
100
120

(b)

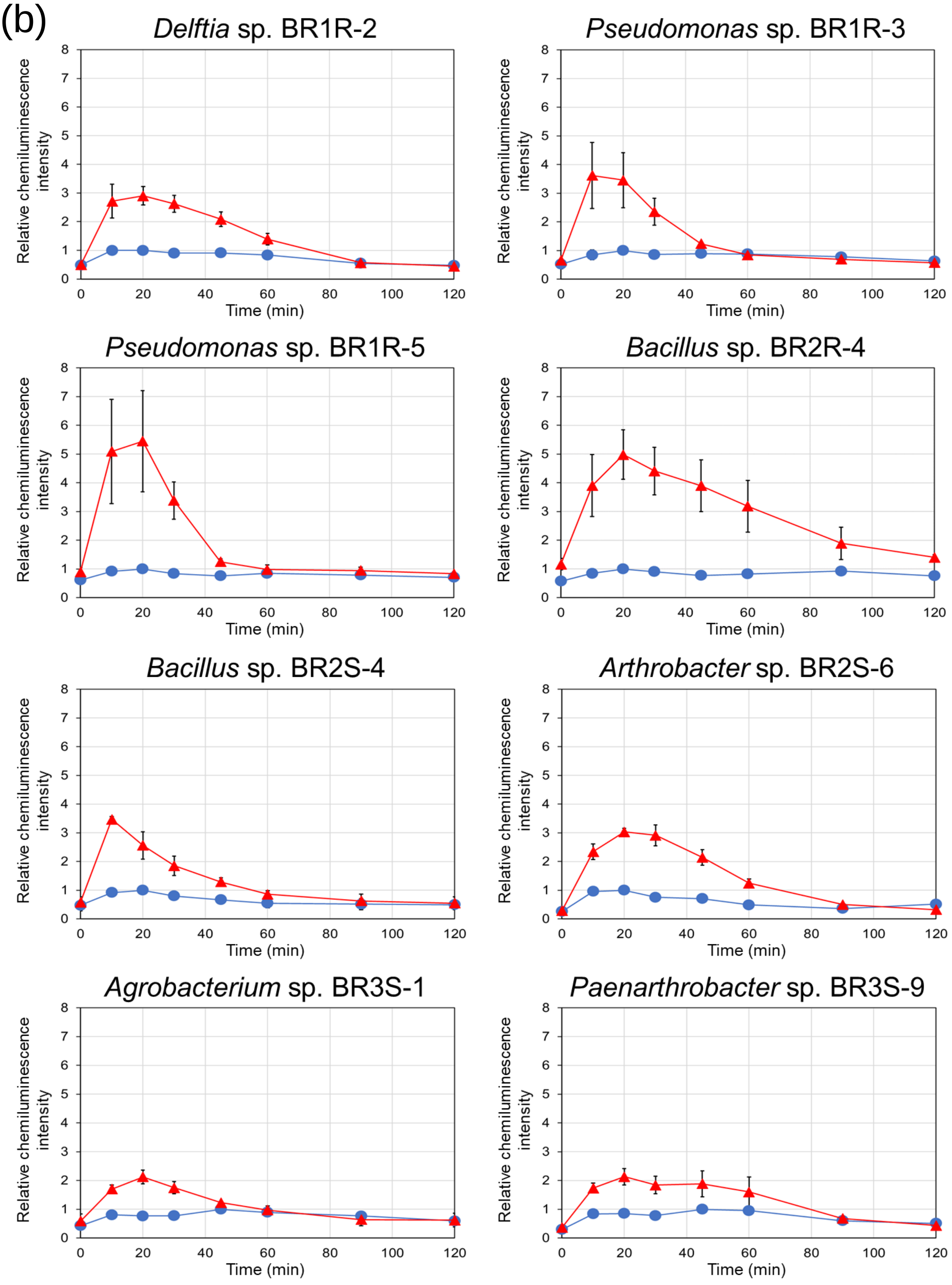

Delftia sp. BR1R-2
Pseudomonas sp. BR1R-3
Pseudomonas sp. BR1R-5
Bacillus sp. BR2R-4
Bacillus sp. BR2S-4
Arthrobacter sp. BR2S-6
Agrobacterium sp. BR3S-1
Paenarthrobacter sp. BR3S-9
Relative chemiluminescence intensity
Time (min)
0
20
40
60
80
100
120
0
1
2
3
4
5
6
7
8

**Figure S3.** Cryptogein-induced ROS production in BY-2 cells co-incubated with bacteria. BY-2 cells were co-incubated with bacteria of each strain (Δ) or mock treatment (only a mixture of the medium and the buffer, ○), and then cryptogein was added. (a) Bacteria that did not enhance ROS production; (b) Bacteria that enhanced ROS production. ROS production was monitored by chemiluminescence. The maximum value of the mock control was expressed as 1.0. Average values ± SE from three independent experiments are presented.

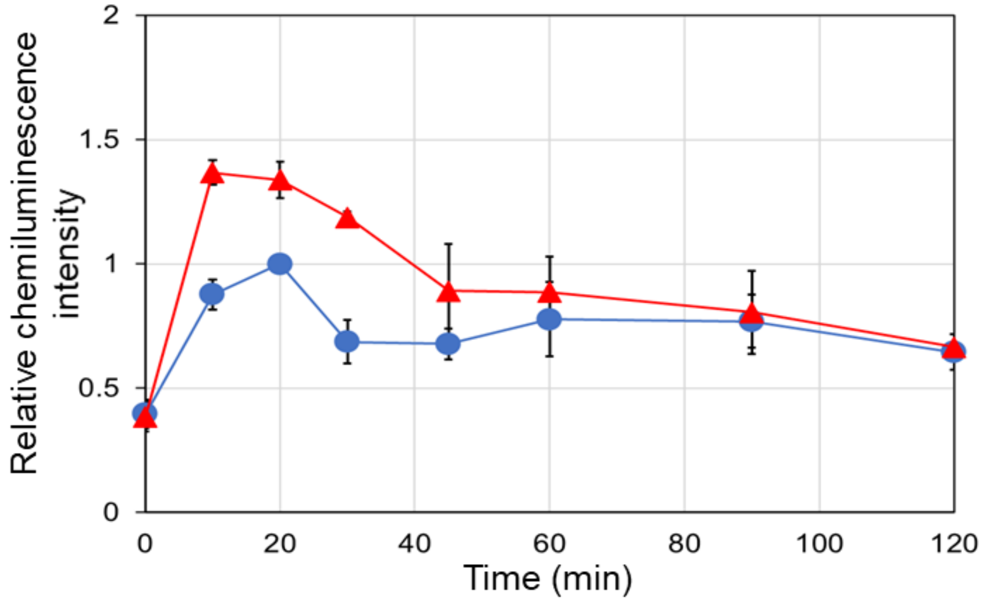


**Figure S4.** Cryptogein-induced ROS production in BY-2 cells co-incubated with *P. phytofirmans* PsJN. BY-2 cells were co-incubated with cells of strain PsJN (Δ) or mock treatment (only a mixture of the medium and the buffer, ○), and then cryptogein was added. The maximum value of the mock control was expressed as 1.0. Average values ± SE from three independent experiments are presented.

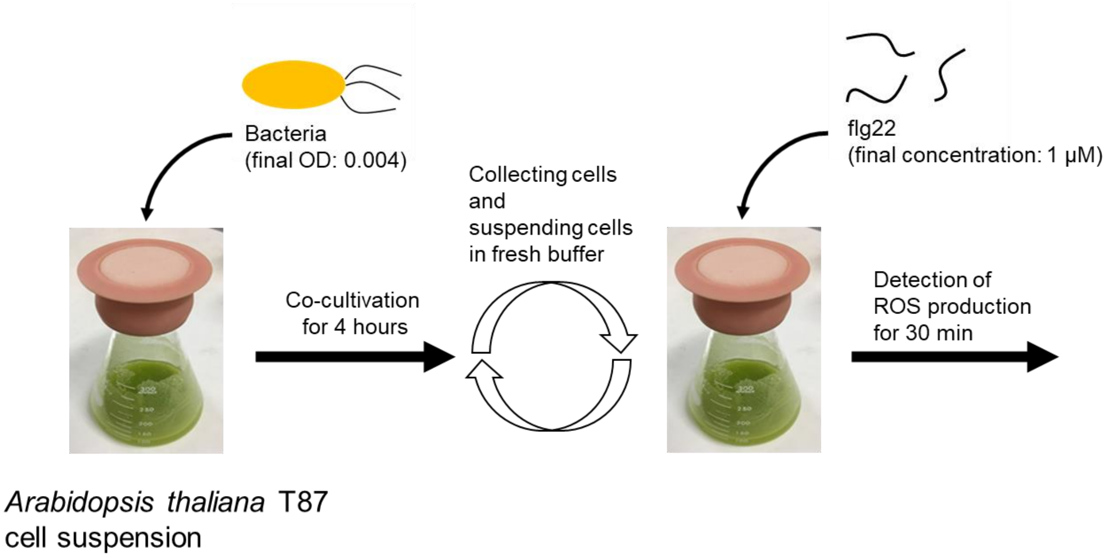


**Figure S5.** Schematic illustration of assays for flg22-induced ROS production in T87 cells co-incubated with bacteria.

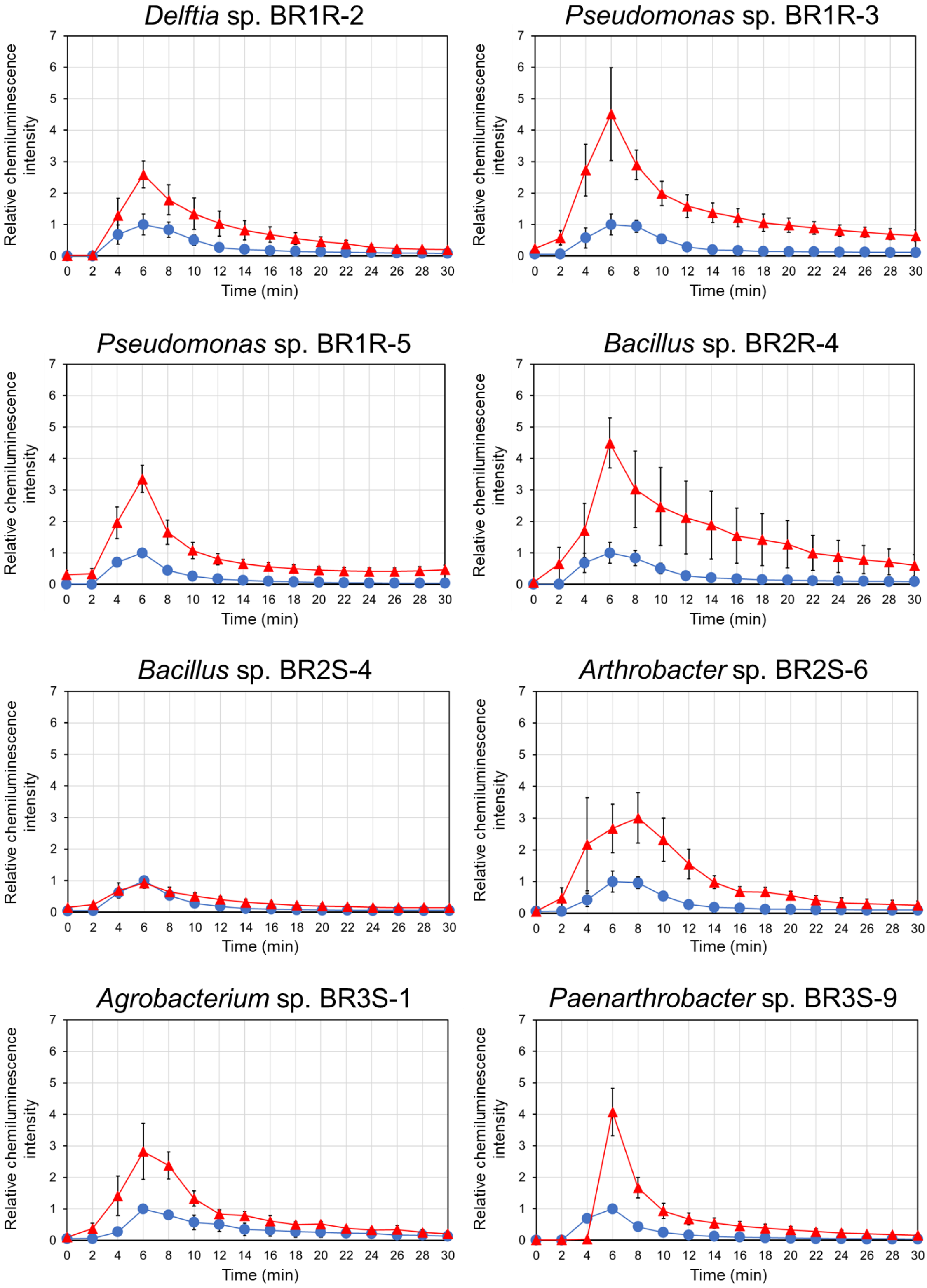
Delftia sp. BR1R-2
Pseudomonas sp. BR1R-3
Pseudomonas sp. BR1R-5
Bacillus sp. BR2R-4
Bacillus sp. BR2S-4
Arthrobacter sp. BR2S-6
Agrobacterium sp. BR3S-1
Paenarthrobacter sp. BR3S-9
Relative chemiluminescence intensity
Time (min)
0
1
2
3
4
5
6
7
0 2 4 6 8 10 12 14 16 18 20 22 24 26 28 30

**Figure S6.** flg22-induced ROS production in T87 cells co-incubated with bacteria. T87 cells were co-incubated with bacteria of each selected strain (Δ) or mock treatment (only a mixture of the medium and the buffer, ○), and then flg22 was added. ROS was monitored by chemiluminescence. The maximum value of the mock control was expressed as 1.0. Average values ± SE from three independent experiments are presented.

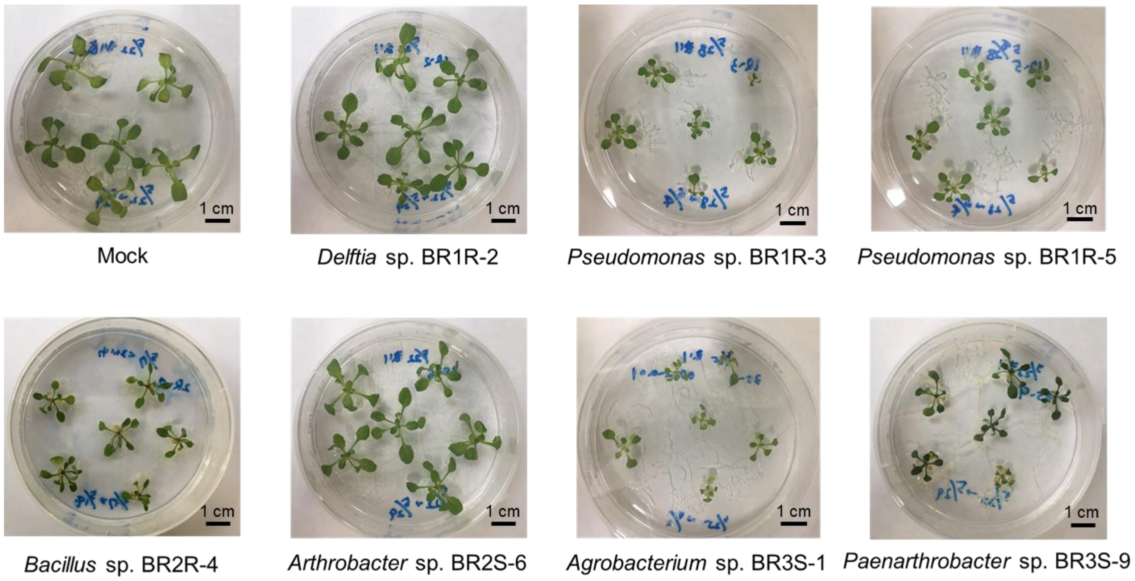


**Figure S7.** *Arabidopsis* plants inoculated with selected bacteria. Plants were inoculated with bacteria of each selected strain or mock treatment (only the medium) by immersing the root tip of 7-day-old seedlings in the bacterial cell culture solution and then cultivated for 7 days.

(a)

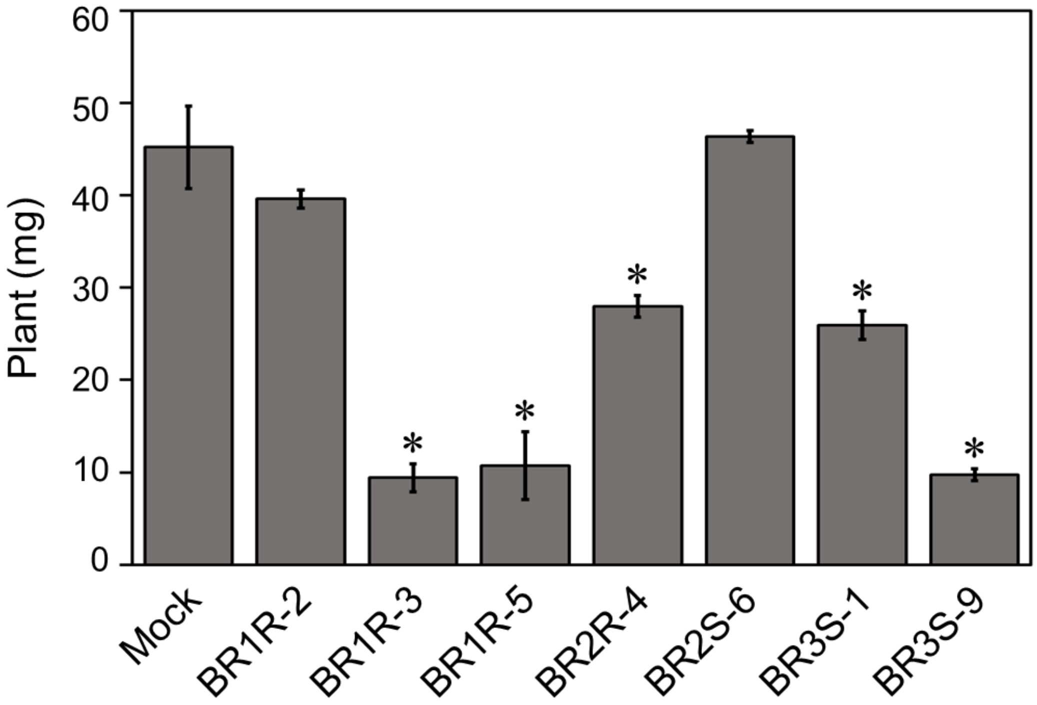
60
50
40
30
20
10
0
Plant (mg)
*
Mock
BR1R-2
BR1R-3
BR1R-5
BR2R-4
BR2S-6
BR3S-1
BR3S-9

(b)

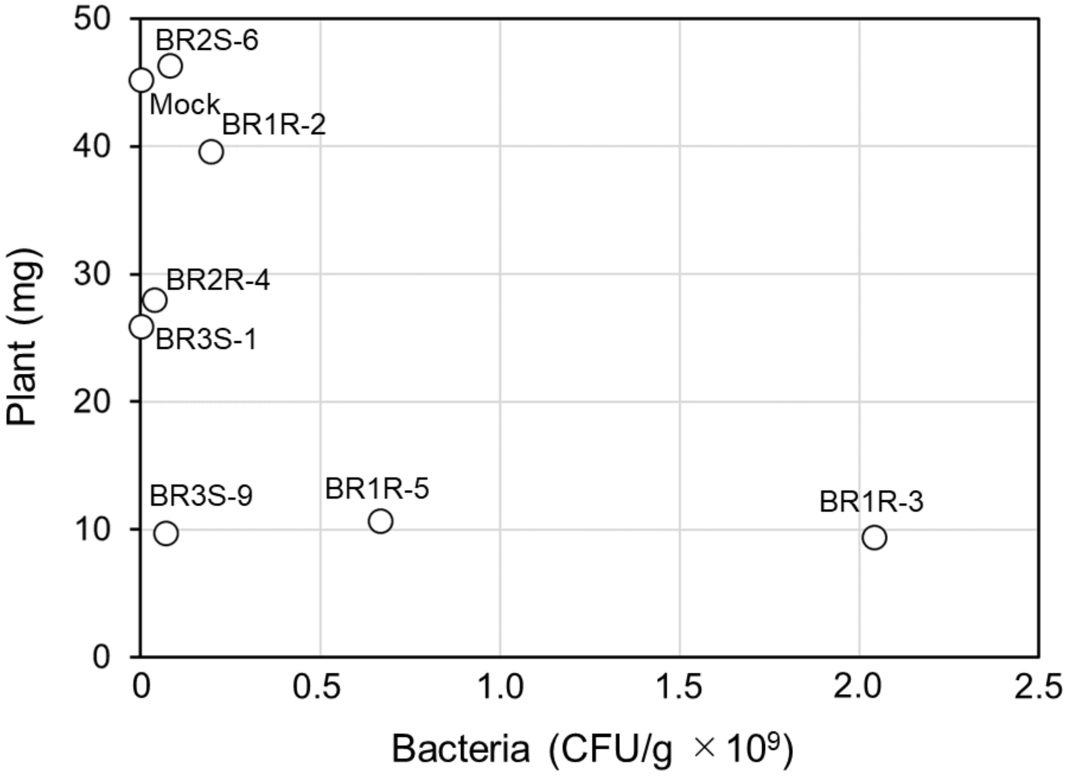
50
40
30
20
10
0
Plant (mg)
BR2S-6
Mock
BR1R-2
BR2R-4
BR3S-1
BR3S-9
BR1R-5
BR1R-3
0
0.5
1.0
1.5
2.0
2.5
Bacteria (CFU/g × 10^9)

**Figure S8.** Fresh weight of *Arabidopsis* plants inoculated with the selected bacteria. Plants were inoculated with bacteria of each selected strain or mock treatment (only the medium) by immersing the root tip of 7-day-old seedlings in the bacterial cell culture solution and cultivated for 7 days. (a) Fresh weight of a plant was determined. Average values ± SE from three independent experiments are presented. Asterisks indicate a significant difference from the mock control based on Student's *t*-test (*, $P<0.05$). (b) Correlation test between the number of bacteria in plants (Fig. 4) and fresh weight of plants (Fig. S8a) is shown ($r = -0.56$).

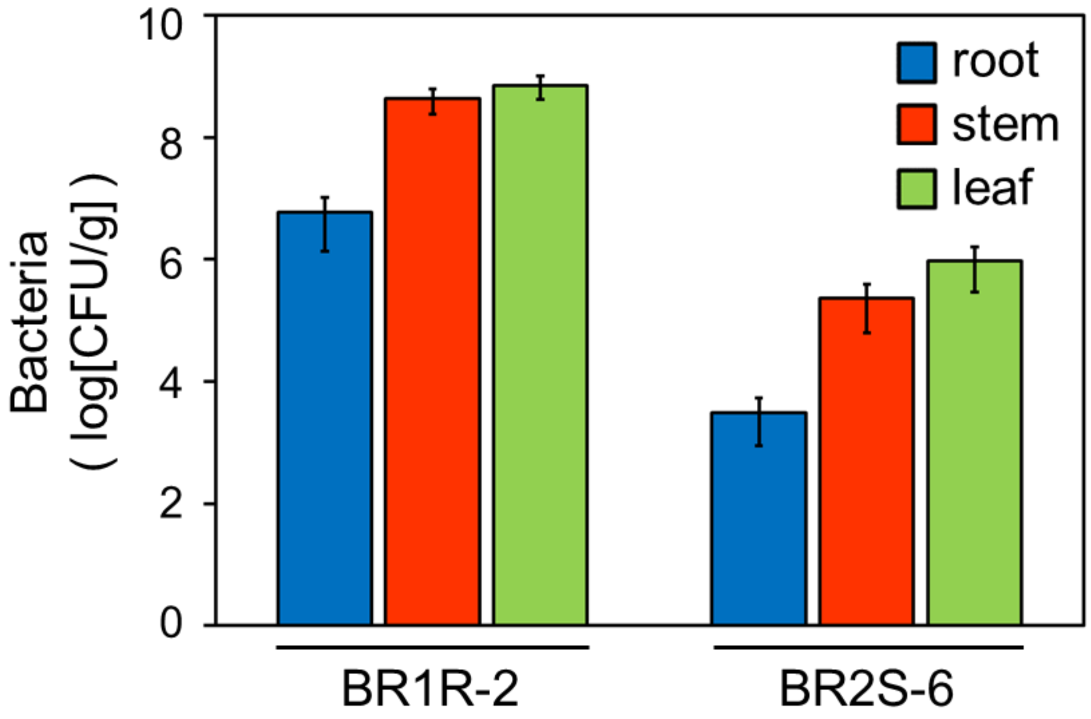


**Figure S9.** Proliferation of strains BR1R-2 and BR2S-6 in *Arabidopsis*. Plants were inoculated with strain BR1R-2 or BR2S-6 by immersing the root tip of 7-day-old seedlings in the bacterial cell culture solution and cultivated for 7 days. After plating extracts of surface-sterilized roots (blue bar), stems (red bar), or leaves (green bar) on medium, colonies formed on the plates were counted. Average values ± SE from three independent experiments are presented.

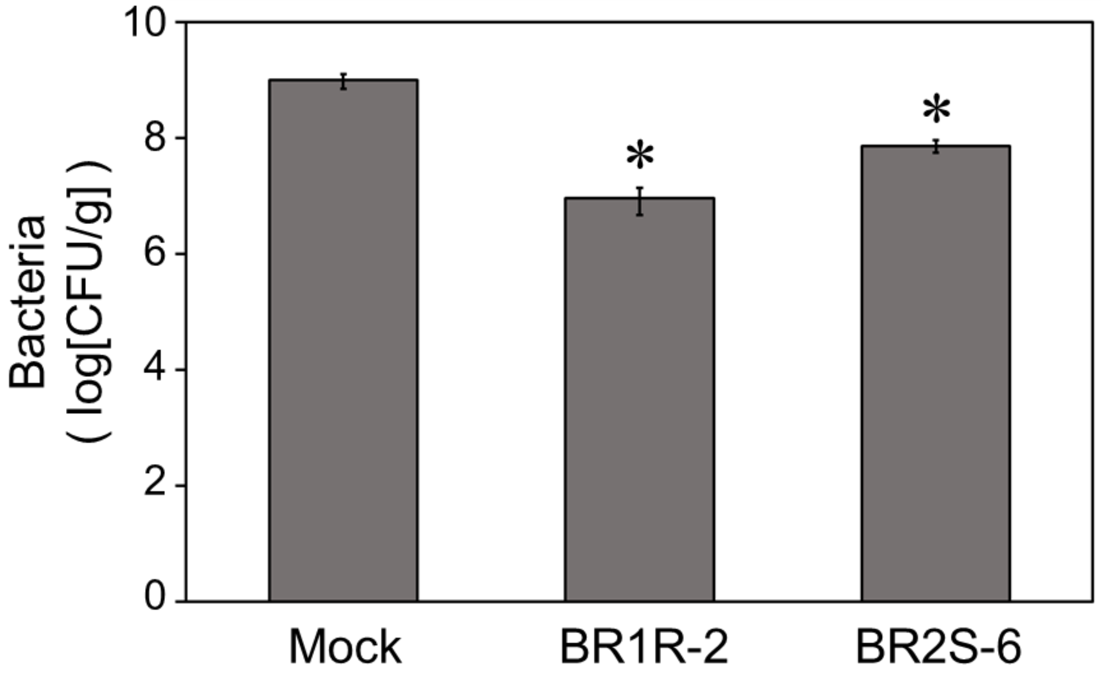


**Figure S10.** Proliferation of *P. syringae* pv. *tomato* DC3000 in *Arabidopsis*. BR1R-2–, BR2S-6–, or mock (only the medium)–treated *Arabidopsis* seedlings were cultivated for 7 days, and the plants were then challenged with *P. syringae* pv. *tomato* DC3000 and cultivated for 3 days. After plating extracts of surface-sterilized aerial tissues of plants on medium, colonies of strain DC3000 formed on the plate were counted. Average values ± SE from three independent experiments are presented. Asterisks indicate a significant difference from the mock control based on Student's t-test (*, P<0.05).
